# Synthesis and Incorporation of a pH‐Responsive Nucleoside Into DNA Sequences

**DOI:** 10.1002/cbic.202500650

**Published:** 2025-10-01

**Authors:** Eric Ogel, Sidney Becker

**Affiliations:** ^1^ Max‐Planck‐Institute of Molecular Physiology Otto‐Hahn‐Straße 11 44227 Dortmund Germany; ^2^ Department of Chemistry and Chemical Biology Technical University Dortmund 44227 Dortmund Germany

**Keywords:** nucleic acids, nucleosides, oligonucleotides

## Abstract

DNA's programable thermodynamics, structural versatility, and ease of synthesis makes it an ideal material for constructing molecular devices. While many biological systems are powered by proton gradients to drive dynamic processes, harnessing pH differences in DNA nanotechnology is possible through pH‐responsive DNA motifs. Existing strategies, however, often depend on strict sequence constraints or nonphysiological pH conditions, limiting their applicability in complex DNA origami structures. In this article, a nucleoside with pH‐sensitive base pairing is developed that reversibly switches its pairing specificity near physiological pH. This unnatural building block is recognized by standard polymerases, and its pairing behavior can be controlled by pH. Characterization of the base pairing properties reveals that duplex stability varies with pH, while canonical sequences remain unaffected. This design enables programable sequence motifs that transition between duplex and single‐stranded DNA in response to pH changes. Our unnatural nucleoside therefore provides a versatile tool for dynamic DNA nanotechnology, with potential applications in DNA nanomachines, biosensing, and targeted drug delivery. Additionally, its physiological p*K*_a_ may enable general acid–base catalysis in ribozymes or DNAzymes, analogous to histidine in protein enzymes.

## Graphical abstract

A pH‐responsive nucleoside is developed that enables a reversible switch in DNA between paired and unpaired states near physiological pH. This design may provide the framework for sequence‐independent, pH programable DNA motifs, thus paving the way for dynamic DNA nanotechnology applications.

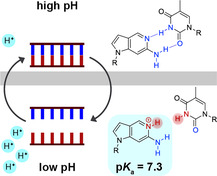

## 1 Introduction

DNA has emerged as a versatile tool to construct molecular devices at the nanoscale, offering precise control over structure, shape, size, and functionality through its programable nature.^[^ ^1^, ^2^ ^–^ ^3^ ^]^ Constructing molecular machines from DNA origami, however, is challenging. Previous work led to multiple designs of rotary assemblies and established a certain level of directed motion.^[^ ^2^ ^,^ ^4^ ^]^ This enabled the imitation of complex biological systems, such as the F_1_F_0_‐ATPase, where directed motion was achieved by Brownian motion with ratchets.^[^ ^4^ ^]^ In nature, proton gradients power a variety of dynamic processes, from ATP synthase function at the nanoscale to cell recognition at the microscale.^[^ ^5^ ^]^ Harnessing pH differences in DNA nanotechnology can be realized using pH‐responsive DNA motifs.^[^ ^6^ ^,^ ^7^ ^]^ Although several DNA folds exhibit such behavior across broad pH ranges, stringent sequence constraints limit their utility.^[^ ^8^ ^]^ DNA triplexes are among the most established motifs, functioning as pH‐dependent switches through Hoogsteen interactions between single‐ and double‐stranded DNA, which can be tuned to operate within specific pH windows.^[^ ^9^ ^,^ ^10^ ^]^ Triplex motifs can be also constructed from peptide nucleic acid utilizing the unnatural nucleobase 2‐Aminopyridine (M, p*K*_a_ ≈ 6.7) to form a pH‐responsive Hoogsteen triplet with a GC base pair upon protonation (M + ·G‐C).^[^ ^11^ ^,^ ^12^ ^]^ The i‐motif offers another pH‐responsive DNA fold, but is also restricted by narrow sequence requirements.^[^ ^13^ ^,^ ^14^ ^]^ In contrast, A^+^‐C wobble interactions offer great sequence flexibility but require acidic conditions near cytosine's p*K*_a_, which disrupts other canonical sequences in complex DNA nanostructures.^[^ ^15^ ^]^ In duplex DNA, one example of a pH‐responsive nucleobase is 3,7‐dideazaadenine with a p*K*_a_ of 8.6.^[^ ^16^ ^]^ It forms a base pair with T when deprotonated (at a pH around 9) and an even more stable base pair with chemically modified 5‐formylcytosine upon protonation.^[^ ^17^ ^]^ It can therefore be used for the detection of epigenetic cytosine modifications in DNA.^[^ ^17^ ^]^

**Figure 1:**
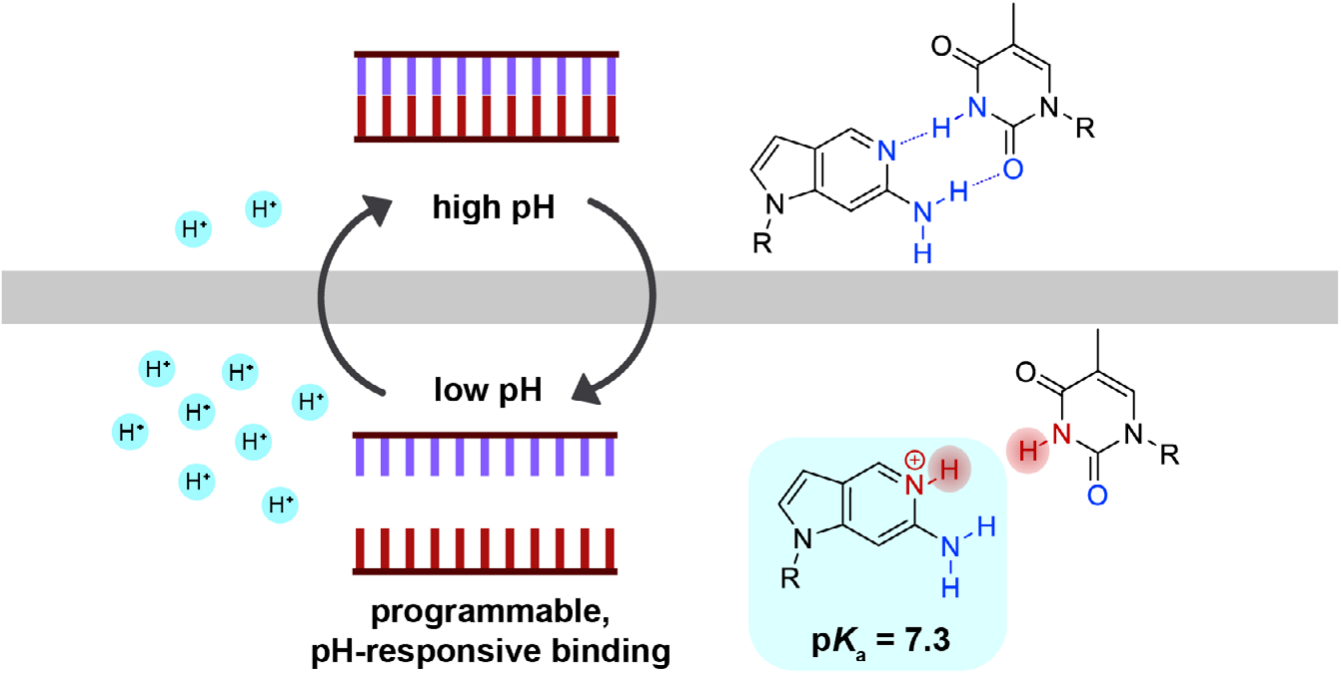
Schematic representation of the pH‐responsive base pair causing denaturation in an environment with low pH.

Here, we present 3,7‐dideaza‐2‐aminopurine (2‐Amino‐DDP) with a p*K*_a_ matching physiological pH, enabling it to control its base pairing properties (**Figure** **1**). The non‐canonical nucleobase undergoes pH‐dependent pairing, where the protonation state determines polymerase incorporation opposite cytosine and thymine. This confirms that base pairing arises from a distinct hydrogen bonding pattern induced by (de)protonation. Characterization of the base‐pairing properties revealed pH‐dependent changes in duplex stability, while canonical sequences remained unaffected. Consequently, our unnatural nucleoside may enable sequence‐independent, pH‐responsive DNA motifs that function orthogonally to natural DNA sequences. This offers a versatile tool to harness pH differences for dynamic DNA nanotechnology applications including DNA nanomachines, biosensing, and drug delivery.^[^ ^18^, ^19^, ^20^ ^–^ ^21^ ^]^

## 2 Results and Discussion

### 2.1 Rational Design of a pH Responsive Nucleoside

**Figure 2:**
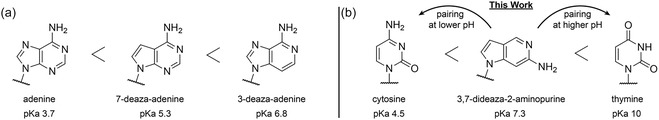
p*K*_a_ values^[^ ^22^ ^]^ of canonical nucleobases and close structural analogs showing higher basicity in deaza‐ and dideaza‐derivatives. a) p*K*_a_ of adenine and its deaza analogs in increasing order. b) pH programmable nature of 3,7‐dideaza‐2‐aminopurine to influence its base pairing properties.

Developing pH‐responsive, orthogonal DNA motifs that preserve canonical sequence integrity requires careful p*K*_a_ consideration. While cytosine is protonated below pH 4.5, thymine deprotonates above pH 10. Both conditions destabilize the DNA duplex and ultimately leads to DNA melting. Thus, an ideal p*K*_a_ for a pH‐responsive nucleoside lies between these two values, creating an optimal window at physiological conditions near pH 7.4. To preserve duplex stability and to ensure enzymatic compatibility, the pH responsive nucleobase should closely resemble canonical bases and ideally engage in programable interactions with standard DNA building blocks. Tuning acid–base properties of heterocycles can be achieved by adjusting their electron density. An attractive approach to increase the p*K*_a_ of nucleobases is to remove nitrogen atoms from the aromatic ring system, as this increases the electron density (**Figure** 2, 1).

Adenine has a p*K*_a_ of 3.7, while 7‐deaza‐adenine has a significantly higher p*K*_a_ of 5.3.^[^ ^22^ ^]^ Removal of both, N3 and N7, further increases the electron density of the heterocycle,^[^ ^22^ ^]^ potentially allowing for protonation under physiological conditions. To demonstrate that the protonation state can influence base pairing, we wanted to design a nucleobase that is capable of pairing with two natural bases. The 2‐aminopurine (p*K*_a _= 3.8) scaffold can theoretically pair with cytosine or thymine, depending on its protonation state at N1. However, its p*K*_a_ is too low achieve protonation under physiological conditions. To enhance the basicity of 2‐aminopurine, we reasoned that 3,7‐dideaza‐2‐aminopurine (2‐Amino‐DDP) would facilitate protonation at higher pH. Protonation at N1 is expected to enable base pairing with cytosine through an additional hydrogen bond, while the deprotonated form of 2‐Amino‐DDP should preferentially pair with thymine (Figure 2b). To test this, we synthesized 2‐Amino‐DDP triphosphate and phosphoramidite for its enzymatic and chemical incorporation into DNA to study its physicochemical properties.

### 2.2 Synthesis of the Triphosphate and Phosphoramidite Analogs

**Scheme 1:**
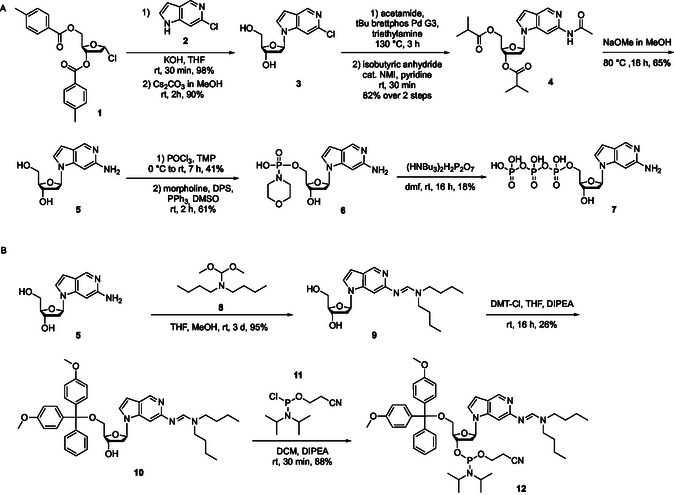
Synthesis of 2‐amino‐DDP nucleoside as triphosphate A) and as DMT‐protected phosphoramidite B).

To test enzymatic incorporation of 2‐Amino‐DDP intoDNA, its triphosphate was synthesized (**Scheme** **1A**). Starting from Hoffer's Chlorosugar **1** and 3,7‐dideaza‐2‐chloropurine **2**, which are both commercially available, the glycosylation and the following deprotection of the hydroxyl groups were conducted as described in the literature^[^ ^23^ ^]^ to give the 2‐Chloro‐DDP nucleoside **3**. The chlorine residue was converted to an amide using a modified Buchwald–Hartwig amidation. Initially, the protocol documented in the literature^[^ ^24^ ^]^ was employed, utilizing *tBu brettphos Pd G3* as preactivated catalyst, Cs_2_CO_3_ as base, tert‐butanol as solvent, and 6.0 eq. of acetamide to give **3b** in 10% yield. To improve the low conversion rate, we optimized the conditions by using acetamide as both a solvent and a reagent, which led to 82% yield. To remove the large amounts of acetamide after the coupling, we protected the 5′‐ and 3′‐hydroxyl groups with isobutyric anhydride, allowing the protected nucleoside **4** to remain in the organic layer while acetamide was extracted through water washes. Subsequent cleavage of the protecting groups under basic conditions provided the unprotected nucleoside **5**. Due to the increased electron density of the heterocycle, the deprotection conditions required elevated temperature to fully remove the amide protecting group. The nucleoside was first converted to its 5′‐monophosphate using POCl_3_ in trimethyl phosphate using a reported procedure.^[^ ^25^ ^]^ The monophosphate was then transformed into the corresponding morpholidate **6**,^[^ ^26^ ^]^ which subsequently reacted with pyrophosphate to yield the 2‐Amino‐DDP triphosphate **7**.

For chemical oligonucleotide synthesis, we prepared the phosphoramidite building block from compound **5**(Scheme 1B). Initial attempts to protect the exocyclic amino group with amide‐based protecting groups worked well, but they proved too stable to be efficiently removed after DNA synthesis. Notably, while pentafluorobenzoyl protecting group could be cleaved from the free nucleoside under mild conditions, its deprotection from the oligonucleotide required harsh conditions and led to DNA degradation. We suspect that enhanced base stacking within the oligonucleotide increases the stability of the protecting group relative to the nucleoside. To overcome this limitation, we employed *N*,*N*‐dibutylformamide dimethylacetal **8** ^[^ ^27^ ^]^ to selectively protect the exocyclic amine of the 2‐Amino‐DDP nucleoside as amidine **9**, which can be readily removed by nucleophiles, even from electron‐rich aromatic heterocycles. The 5′‐hydroxyl group was then protected with a DMT group to yield intermediate **10**.^[^ ^28^ ^]^ Finally, treatment with 2‐cyanoethyl *N*,*N*‐diisopropylchlorophosphoramidite **11** afforded the fully protected phosphoramidite building block **12** for solid phase DNA synthesis.^[^ ^29^ ^]^

### 2.3 Testing pH Sensitivity upon Polymerase Incorporation

**Figure 3:**
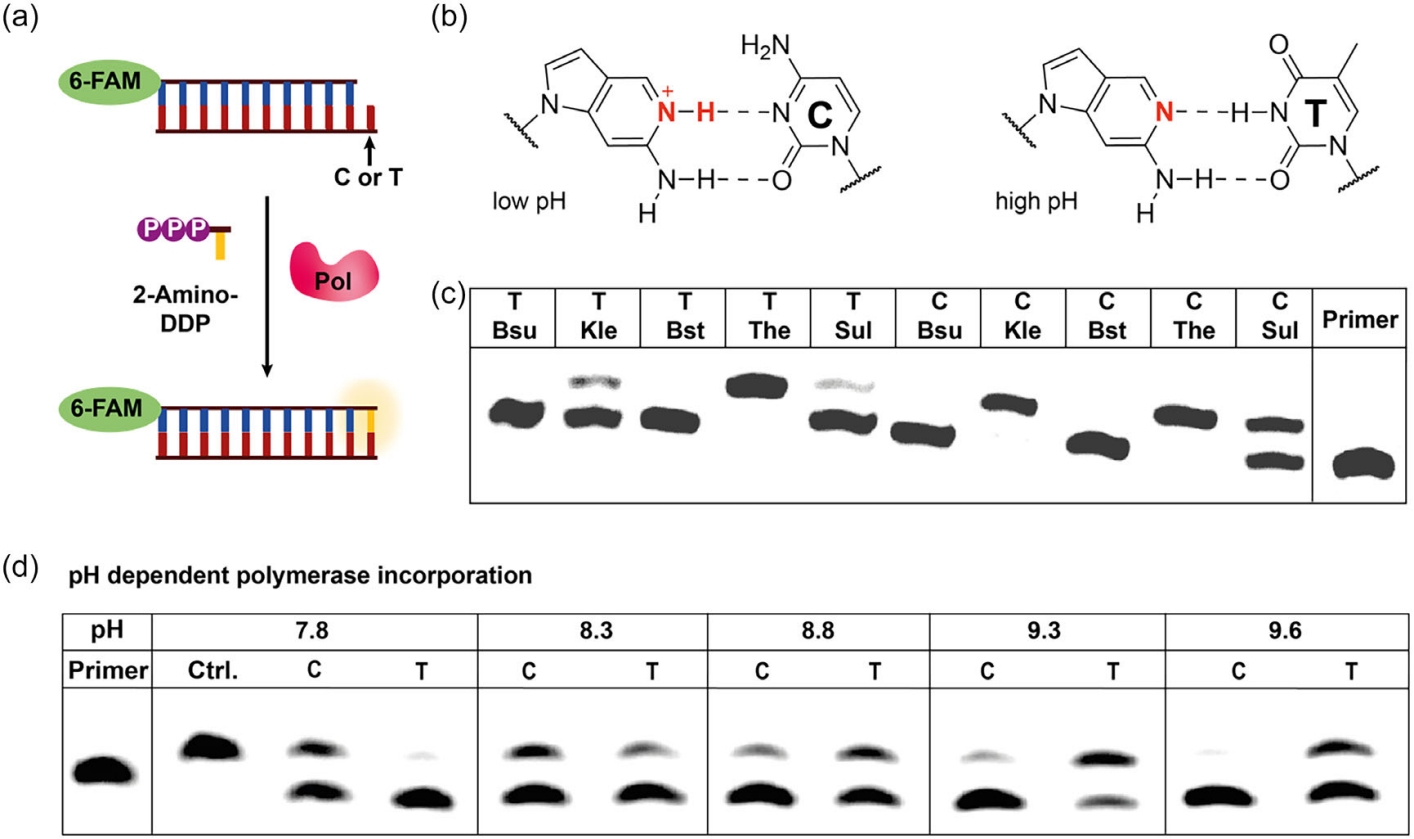
a) Schematic illustration of the single nucleotide incorporation with a DNA polymerase. b) pH‐dependent base pairing of 2‐Amino‐DDP with cytosine and thymine. c) Urea‐PAGE of the enzymatic incorporation of 2‐Amino‐DDP with different polymerases. Conditions: 0.5 µM template, 0.25 µM primer, 100 µM dNTP, 0.05 units µL^−^^1^ polymerase, 10 min. Temp: 60 °C for Bst, Kle and Sul, 37 °C for Bsu and Kle. Bsu (Bsu DNA Polymerase, Large Fragment), Kle (Klenow‐Fragment), Bst (Bst DNA Polymerase, Large Fragment), The (Therminator DNA Polymerase), Sul (Sulfolobus DNA Polymerase IV). d) Urea‐PAGE of the enzymatic and pH‐dependent incorporation of 2‐Amino‐DDP with Thermo Sequenase DNA Polymerase (Cytiva), conditions: 0.5 µM template, 0.25 µM primer, 25 µM dNTP, 0.05 units µL^−^^1^ polymerase, 30 mM Tris‐HCl, 7.5 mM MgSO_4_, 60 °C, 10 min.

To demonstrate the pH‐responsive behavior of 2‐Amino‐DDP triphosphate, we conducted polymerase incorporation assays using single‐nucleotide extension reactions (**Figure** **3**a,b). Due to the absence of N3, which reduces electron density in the minor groove, we first assessed whether exonuclease‐deficient polymerases could accept this nucleotide under their standard buffer conditions. Notably, the unnatural triphosphate was incorporated by several DNA polymerases, including KlenTaq, Thermo Sequenase, Klenow, Therminator, and Sulfolobus, while the Bsu and Bst polymerase showed no incorporation activity (Figure 3c). Next, we investigated whether incorporation opposite T and C could be pH‐controlled. At lower pH, 2‐amino‐DDP was preferentially incorporated opposite cytosine by Thermo Sequenase, whereas at higher pH, incorporation shifted to favor thymine in the template strand (Figure 3d and S1, Supporting Information). Similar results were also obtained using KlenTaq polymerase (Figure S2 and S3, Supporting Information). As a control, we tested the enzymatic incorporation of commercially available 2‐aminopurine (2AP) triphosphate. Despite its reported mild mutagenicity,^[^ ^30^ ^]^ no significant incorporation opposite C was observed at any tested pH (Figure S4, Supporting Information). This supports our hypothesis that the pH responsiveness of the 2‐Amino‐DDP building block arises from its increased basicity, resulting from the substitution of two nitrogen atoms with carbon in the heterocycle. To our knowledge, this is the first report of pH‐dependent base pairing specificity in which a nucleobase switches its pairing preference between two canonical bases during enzymatic incorporation.

To assess whether the unnatural base pair induced polymerase stalling, we initially performed the extension of a 30‐mer primer with 2‐Amino‐DDP triphosphate using a 49‐mer DNA template, which resulted in two incorporations opposite T as expected. The calculated product mass of 10,296.849 Da is in agreement with the deconvoluted experimental mass of 10,296.861 Da (Figure S12, Supporting Information). Additionally, 3′‐A‐tailing of the template was observed. Subsequent addition of all remaining dNTPs enabled full primer elongation without stalling, plus 3′‐A‐tailing at the end. The expected mass of 15,904.786 Da was confirmed by the deconvoluted experimental mass of 15,904.762 Da (Figure S13, Supporting Information). As our building block is readily accepted by polymerases it may be used for in vitro evolution studies for example to create DNAzymes. The physiological p*K*_a_ of our nucleobase might allow for general acid–base catalysis analogous to histidine in protein enzymes.

### 2.4 Biophysical Characterization of Base Pairing

**Figure 4:**
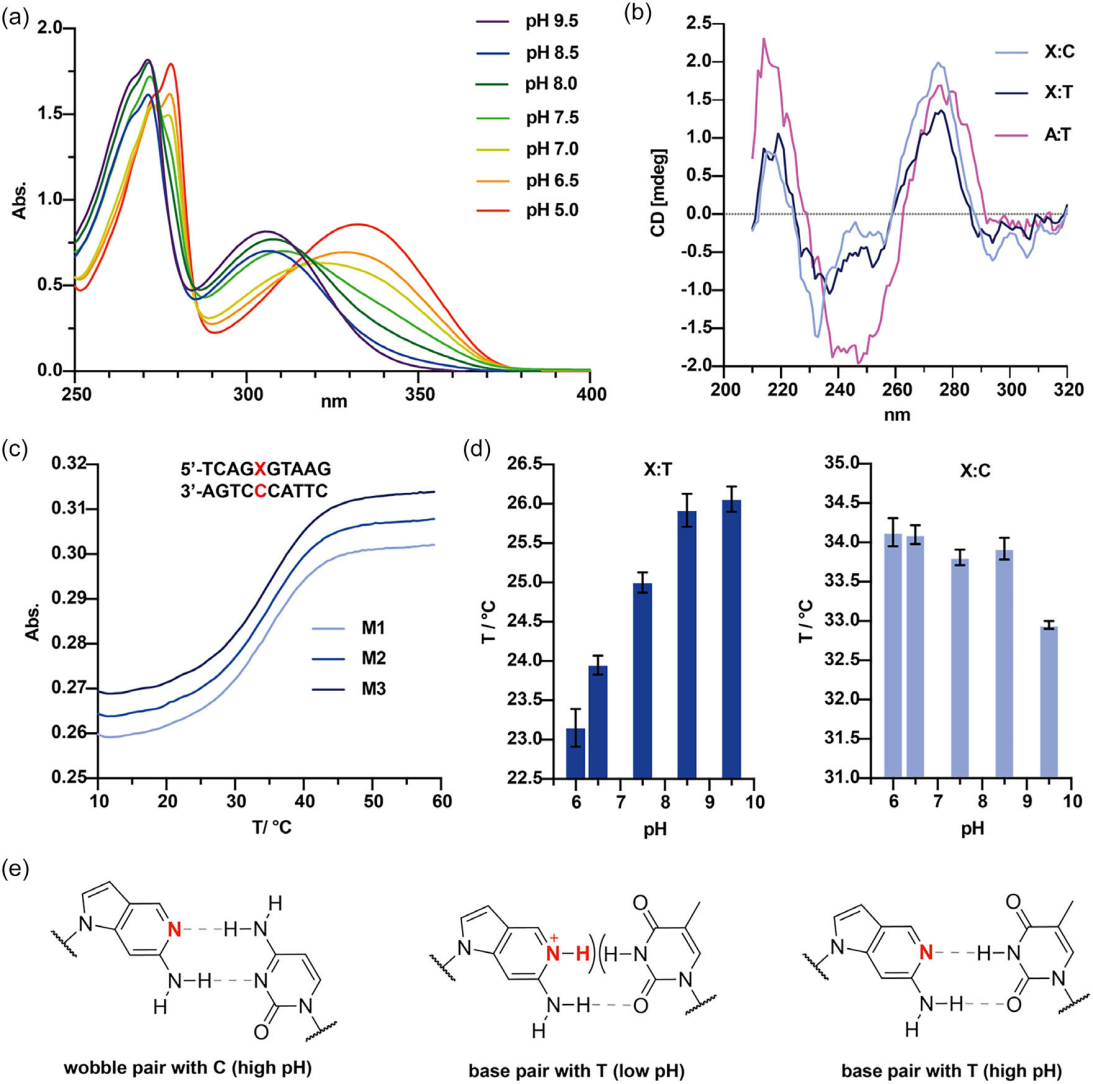
a) UV–Vis absorption spectrum of 2‐Amino‐DDP nucleoside in aqueous buffers at pH values ranging from 5.0 to 9.5. b) CD spectra of the 10‐mer duplex with C‐ or T‐template and AT as control, at a pH of 6.5, measured at 20 °C. The displayed results are the mean of three consecutive measurements. c) Three melting curves of the 10‐mer duplex ’XC’ at pH 6.5, determined by the absorption difference between 260 and 420 nm and presented as illustrative examples. d) Melting points at five different pH values had been measured by the UV absorption change at 260 nm. The results from the duplex in which 2‐Amino‐DDP forms a base pair with T are shown on the left, and with C on the right. e) Watson–Crick‐base pairing of 2‐Amino‐DDP pair with T and C, and potential wobble pair with C.

To further characterize 2‐Amino‐DDP base pairing, we performed physicochemical analysis. First, we determined the p*K*_a_ of 2‐Amino‐DDP to assess whether its acid–base properties align with the pH‐dependent behavior observed during polymerase incorporation. To this end, we measured the UV/Vis absorption spectrum of the nucleoside in aqueous buffer across a pH range of 5.0–9.5. Protonation at the aromatic nitrogen leads to a distinct shift in the absorption spectrum with decreasing pH (**Figure** **4**a) indicating a change in the electronic properties of the nucleobase.

In addition to an absorption maximum at 260–270 nm, which is characteristic of aromatic compounds, a second peak emerges at around 310 nm within the basic pH range. As the pH of the buffer decreases, this second peak experiences a shift toward longer wavelengths, reaching 335 nm at pH 5.0. A plot of the absolute difference in absorption versus pH allowed us to determine the p*K*_a_. The difference in absorption at 340 nm was used to calculate the p*K*_a_ value using nonlinear regression, with a method described by Dardonville and colleagues^[^ ^31^ ^]^ to provide the p*K*_a_ (2‐Amino‐DDP) of 7.3 ± 0.2. The p*K*_a_ closely matches physiological pH, potentially enabling to harness biological proton gradients to power molecular machines built from DNA nanostructures. To investigate the secondary structures and determine the conformation of double‐stranded DNA, CD spectra of a 10‐mer duplex with C or T in the template strand as well as the nonmodified AT 10‐mer were measured (Figure 4b). The presence of a strong positive peak at 275–280 nm and a negative peak at 245–250 nm is indicative of a B‐DNA structure.^[^ ^32^ ^]^ The spectrum best matches that of a B‐DNA in terms of the relative position of the maxima and minima. The intensity of the spectrum for the base pair with T is slightly lower than that for the base pair with C. This may be due to partial protonation of 2‐Amino‐DDP, which results in repulsion at the Watson–Crick site and therefore influences the duplex structure.

Since enzymatic incorporation alone does not allow direct conclusions about the thermodynamic stability of the new pH‐responsive base pair, we performed melting curve analysis to assess whether its p*K*_a_ influences duplex stability under pH variations. The oligonucleotide TCAGXGTAAG (X = 2‐Amino‐DDP) was synthesized using compound **12** and annealed with its complementary strand CTTACCCTGA yielding sigmoidal melting curves upon heating (Figure 4c). Nonlinear regression was used to fit the curves and determine melting temperatures across different pH conditions, allowing us to assess the pH sensitivity of the modified base pair (Figure 4d). We found that the 2‐Amino‐DDP building block does not exhibit significant pH sensitivity in duplex stability when paired with cytosine. Only a slight decrease in thermal stability was observed from 34.1 °C at pH 6.0 to 32.9 °C at pH 9.5 (Figure 4d). One possible explanation is the formation of a wobble base pair with cytosine upon deprotonation (Figure 4e). As a reference, we analyzed the same oligonucleotide with a canonical A:T base pair at the same position. Its melting temperature was slightly higher, ranging from 34.8 °C at pH 6.0 to 35.5 °C at pH 9.5, indicating no pH responsiveness, as expected. The 2‐Amino‐DDP:T base pair on the other hand exhibits clear pH responsiveness, with melting temperatures increasing from 23.2 °C at pH 6.0 to 26.1 °C at pH 9.5 (Figure 4c). Unlike pairing with cytosine, this base pair cannot form a stabilizing wobble interaction upon protonation. The 2‐Amino‐DDP:T pair also displays significantly lower melting temperatures compared to the corresponding 2‐Amino‐DDP:C base pair. This reduced thermal stability may result from perturbations in duplex dynamics and structure, similar to effects observed with the 2AP:T pair, which is known to influence the lifetimes of up to three adjacent base pairs in both directions. While 2AP does not induce premelting, it lowers the global melting temperature of the duplex.^[^ ^30^ ^]^ A similar effect is presumed for our 2‐Amino‐DDP:T pair. Another possible explanation is that pairing with thymine may introduce unfavorable secondary hydrogen bonding interactions in the Watson–Crick site, whereas pairing with cytosine in the Watson–Crick geometry allows for favorable secondary H‐bonding interactions (Figure 4e). Importantly, the markedly reduced melting temperature of the 2‐Amino‐DDP:T pair offers a unique advantage: it allows for selective sequence targeting, while leaving canonical sequences with inherently low melting temperatures unaffected.

## 3 Conclusion

In summary, we have developed a programable nucleoside with pH‐sensitive base pairing that enables a reversible switch in pairing specificity near physiological pH. This design opens the possibility for sequence‐independent, pH‐responsive DNA motifs that operate orthogonally to natural DNA sequences, paving the way for dynamic applications in DNA nanotechnology. Our findings lay the groundwork for DNA origami structures capable of harnessing pH differences to function as truly dynamic systems for use in molecular machines, biosensing, or targeted drug delivery.

## Supporting Information

Supporting Information for this manuscript includes methods, supplementary figures, additional gel images, MS‐ and NMR‐spectra, and is provided as a pdf file.

## Conflict of Interest

The authors declare no conflict of interest.

## Author Contributions

**Sidney Becker**: conceptualized the project. **Eric Ogel**: conducted the experiments and data analysis. Sidney Becker and Eric Ogel: wrote the manuscript text and prepared the figures.

## Supporting information

Supplementary Material

## Data Availability

The data that support the findings of this study are available in the supplementary material of this article.
