# Supplementary material for: Synthesis and Incorporation of a pH‐Responsive Nucleoside Into DNA Sequences
Source: Chembiochem. 2025 Oct 1;26(21):e202500650. doi: 10.1002/cbic.202500650 (PMC12596921; doi:10.1002/cbic.202500650)
Supplement: Supplementary file 1 — Supplementary Material [file CBIC-26-e202500650-s001.pdf]

Supporting information to: Synthesis and incorporation of a pH-responsive nucleobase into DNA sequences

Eric Ogel[a], Sidney Becker[a]*

[a]*Max-Planck-Institute of Molecular Physiology, Otto-Hahn-Straße 11, 44227 Dortmund, Germany*

* *e-mail: Sidney.becker@mpi-dortmund.mpg.de*

General Information

Unless otherwise specified, all commercially available reagents and solvents were utilized without undergoing additional purification. Solvents used for flash column chromatography were of laboratory-grade quality. Dry solvents were procured from Fischer Scientific, Acros, or VWR, and were used without additional processing. The commercially available oligonucleotides were purchased from Sigma-Aldrich / Merck, in HPLC-purified quality. They were ordered dry, and diluted with Milli-Q-water to produce 100 µM solutions. Purification of crude compounds was achieved through silica gel column chromatography (Merck 60, particle size 0.040-0.063 mm) using indicated solvents.

Analytical thin-layer chromatography (TLC) was conducted on silica-coated aluminum plates (Merck 60 F254) and visualization of synthetic products was achieved through UV irradiation (254 nm and/or 356 nm) and potassium permanganate staining (1.5 g $KMnO_4$, 10 g K_2CO_3 in 1.25 mL of 10% aq. NaOH and 200 mL water). Analytical ultra-high-performance liquid chromatography-mass spectrometry (uHPLC-MS) and liquid chromatography-mass spectrometry (LC-MS) were performed and evaluated using an Agilent 1290 Infinity system equipped with a mass detector (column: Zorbax Eclipse C18 Rapid Resolution 2.1x50 mm 1.8µm, flow rate: 0.5 mL/min) or an Agilent Infinity HPLC system with a 3x50 mm, 1.8 µm Macherey-Nagel Nucleodur C18 Gravity column (flow rate: 0.5 mL/min). For LC-MS analysis, a gradient was applied, starting with 10% acetonitrile (incl. 0.1% formic acid) in water (incl. 0.1% formic acid) and progressing to 100% acetonitrile (incl. 0.1% formic acid).

Nuclear magnetic resonance (NMR) spectra were recorded using one of the following spectrometers: Bruker AV 400 Avance III HD (NanoBay), Bruker AV 500 Avance III HD (Prodigy), Bruker Avance NEO – 500 MHz, Bruker AV 600 Avance III HD (CryoProbe), or a Bruker AV 700 Avance III HD (CryoProbe) spectrometer. Chemical shifts are reported in parts per million (ppm) with reference to the deuterated solvent. Multiplicities are abbreviated as follows: s = singlet, d = doublet, t = triplet, dd = double doublet, and m = multiplet. Coupling constant values are expressed in Hertz. Signals were assigned to their

corresponding hydrogens or carbons based on 2D NMR correlations ($^{1}H/^{1}H$ COSY, $^{1}H/^{13}C$ HSQC, $^{1}H/^{13}C$ HMBC). For phosphorus-containing compounds, ^{31}P NMR analysis was conducted as well.

High-resolution mass spectrometry (HRMS) in positive mode was performed using an LTQ Orbitrap mass spectrometer with electron spray ionization coupled to an Accela HPLC-system (HPLC column: Hypersyl GOLD, 50 mm x 1 mm, particle size 1.9 µm).

HRMS in negative mode (for phosphates and oligonucleotides) was measured using an Orbitrap Exploris 120 ESI-MS (Thermo Fisher Scientific). The HPLC was used with a flow rate of 0.20 mL/min in a solvent system of solvent A (50 mM HFIP and 15 mM TEA in water at pH 9.00) and solvent B (methanol) at 80 °C on a DNAPac RP 4 µm column (2.1 × 100 mm, Thermo Fisher Scientific).

Solid phase DNA synthesis

Oligonucleotides were synthesized on CPG (Controlled Pore Glass) resin with 500 Angstrom Pore Size and 25-35 µmol/g loading from Applied Biosystems™, following modified DNA standard methods. Detritylation was achieved with dichloroacetic acid in DCM (3% v/v) for 45 seconds. For coupling, 100 µL of 100 mM phosphoramidite solution in acetonitrile was activated with 250 µL of 0.25 M ethyl-thiotetrazole (ETT) in acetonitrile. The coupling time was 30 seconds for canonical nucleosides and 10 min for 2-Amino-DDP. Capping was achieved with a 1:1 (v/v) mixture of acetic anhydride in acetonitrile and *N*-Methylimidazole in acetonitrile for 45 seconds. Oxidation was performed with 20 mM I_2 in pyridine/water (9:1 v/v) for 60 seconds. All sequences were synthesized with final trityl-deprotection (DMT-off). Cleavage from the solid support and deprotection was achieved after treatment with 25% ammonia solution for 6 h at 60 °C. HPLC purification was conducted using a Thermo Scientific™ DNAPac™ RP-HPLC-C18 column with 100 mM aqueous TEAA buffer (A) and a mixture of 80% acetonitrile and 20% 500 mM aqueous TEAA buffer (B). The oligonucleotides were analyzed by LC/MS using an Orbitrap Exploris 120 Mass Spectrometer.

For oligonucleotide TCAGXGTAAG (with X=2-Amino-DDP), 128 µg (41.6 nmol) were isolated.

General procedure for triphosphate purification

The initial purification process, carried out using high-performance liquid chromatography (HPLC), involved the use of an ion exchange column with a Tris-HCl buffer and an aqueous, 1.25-M NaCl solution. The process enabled the removal of byproducts, such as the nucleoside monophosphate, while the

nucleoside diphosphate remained unseparated from the triphosphate. Following this initial purification, an additional HPLC purification was conducted, using a Thermo Scientific™ DNAPac™ RP-HPLC-C18 column with 100 mM aqueous TEAA buffer (A) and a mixture of 80% acetonitrile and 20% 500 mM aqueous TEAA buffer (B). The triphosphate was isolated, dried under high vacuum, and stored at -70 °C.

Primer extension experiments

To test the incorporation of a single 2-Amino-DDP, a 24-mer primer with the fluorophore 6-FAM attached at the 5'-end was annealed to a 25-mer template with a C or T overhang at its 5'-end. The sequences are:
Primer: (FAM)-GTTTTGGCTACCTGTTACTAAGCA
Template: **T**TGCTTAGTAACAGGTAGCCAAAAC or **C**TGCTTAGTAACAGGTAGCCAAAAC

For the 30-mer primer with biotin (Btn) attached at the 5'-end that was annealed to a 49-mer template the sequences are:
Primer: (Btn)-CGGGCGGACCAGAACCCTTGAGCACAGAAA
Template: CTTCGGCTAACTCGACGTTTTTCTGTGCTCAAGGGTTCTGGTCCGCCCG

Template (2 eq.) and primer (1 eq.) were annealed by incubating them together at 95 °C for 5 min and cooling down to 10 °C over 30 min. If not stated otherwise, primer extension reactions were performed in a total reaction volume of 20 µL containing 0.25 µM 6-FAM primer, 0.5 µM template, 25 to 50 µM dNTP, 0.1 units/µL polymerase, 20 mM Tris-HCl, 10 mM $(NH_4)_2SO_4$, 10 mM KCl and 5 mM $MgSO_4$. The mixtures were incubated at 37 °C (for Bsu and Kle) or at 60 °C (all other tested polymerases) for the stated times. The resulting oligonucleotide strands were separated using Urea-PAGE and detected by the fluorescence signal caused by 6-FAM.

For the extension of a 49-mer template with a 5'-biotin-modified 30-mer primer, in the first step, 2-Amino-DDP triphosphate was added and successfully incorporated two times (Figure S12). The conditions are: 100 µL total reaction volume, 5.0 µM template, 2.5 µM primer, 20 µM dNTP, 0.05 units/µL Therminator DNA polymerase, 20 min. Temp: 75 °C. In the next step, all other dNTPs were added and the residual 17 units were added. The conditions are: 100 µL total reaction volume, 5.0 µM template, 2.5 µM primer, 20 µM dNTP, 100 µM each of dATP, dGTP, dCTP, dTTP, 0.05 units/µL Therminator DNA polymerase, 40 min. Temp: 75 °C.

Gel preparation

To prepare the Urea-PAGE gels, ROTIPHORESE® Sequenziergel concentrate (240 mL), ROTIPHORESE® Sequenziergel diluent (30 mL) and ROTIPHORESE® Sequenziergel buffer concentrate (30 mL) were mixed. The Concentrate contains 237.5 g/L of acrylamide, 12.5 g/L of methylene bisacryl-amide, and 7.5 M urea in aqueous solution. The Diluent contains 7.5M urea in aqueous solution. The Buffer contains 0.89 M tris-borate 20 mM EDTA buffer at pH 8.3 (10X TBE) and urea. This leads to a final monomer concentration of 20% which was suitable to separate DNA fragments of around 20-30 nucleotides length. APS (2.4 mL of a 10% aqueous solution) and TEMED (120 µL) were added and the gels casted, the comb was inserted and it was left for polymerization for at least 60 min. The electrophoresis unit was filled with TBE-buffer, and the gel was heated by a pre-run for 30 min, before the samples were loaded and separated at 800 W for 3:00 h. Readout of the fluorescent bands was performed with an imager (FX, Bio-Rad).

Additional single nucleotide incorporation experiments

2-Aminopurine was incorporated under the same conditions with the KlenTaq polymerase as control, and no incorporation opposite C occurred at any tested pH (Figure S1).

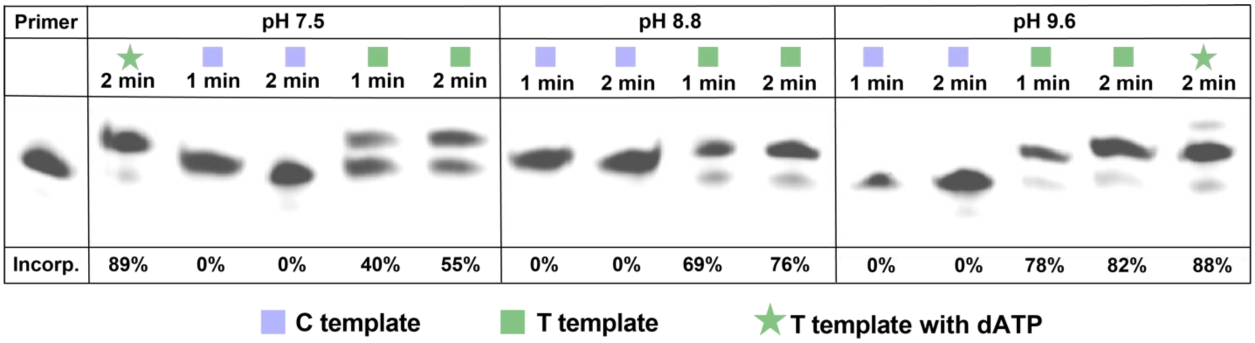

Figure S1: Urea-PAGE of the incorporation of 2-AP with KlenTaq. Conditions: 0.5 µM template, 0.25 µM primer, 25 µM dNTP, 0.05 units/µL polymerase, 25 mM Tris-HCl, 40 mM KCl, 5 mM $MgSO_4$, 60 °C. The experiments where dATP was incorporated opposite a T template were conducted as positive control.

2-Amino-DDP triphosphate was incorporated with Thermo Sequenase under different conditions, including higher polymerase concentration, trying to achieve full incorporation.

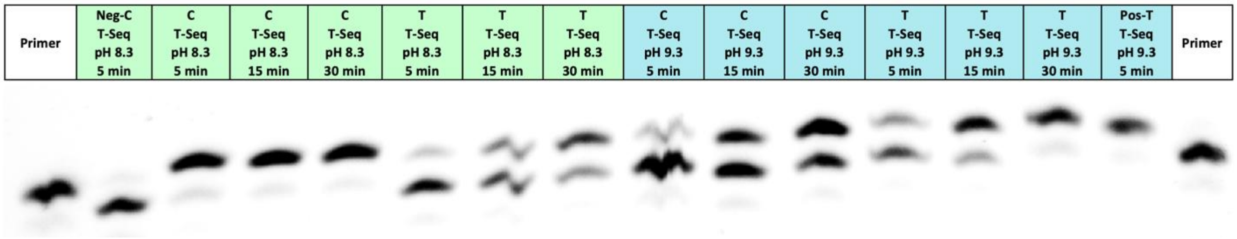

Figure S2: Urea-PAGE of the incorporation of 2-Amino-DDP triphosphate with Thermo Sequenase™ DNA Polymerase (Cytiva), conditions: 0.5 µM template, 0.25 µM primer, 25 µM dNTP, 0.10 units/µL polymerase, 30 mM Tris-HCl, 7.5 mM $MgSO_4$, 60 °C. Neg-C (negative control): no dNTP added. Pos-C (positive control): 25 µM dATP as only dNTP.

With 0.10 units/µL polymerase, the incorporation opposite C was essentially complete after 5 min at pH 8.3, while incorporation opposite T was significantly slower. At pH 9.3., full incorporation opposite T was achieved after 30 min, while incorporation opposite C was significantly slower.

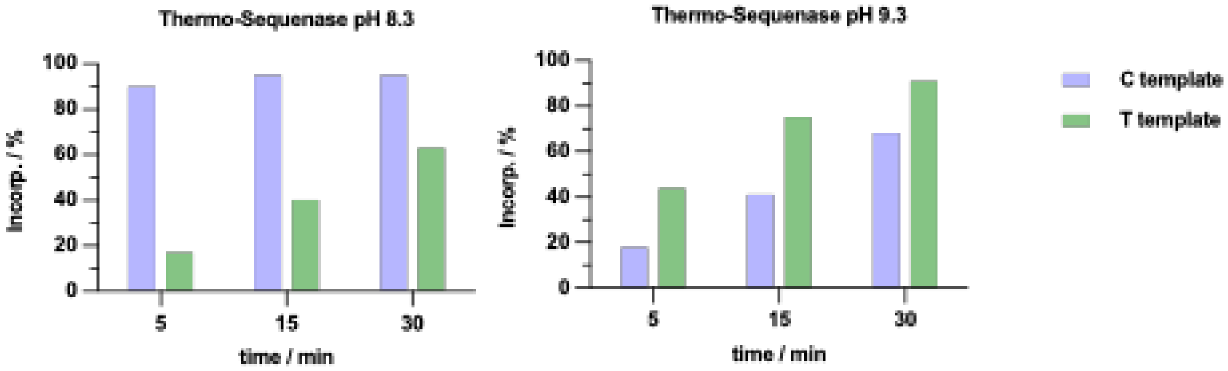

Figure S3: Time-dependent incorporation of 2-Amino-DDP triphosphate with Thermo Sequenase™ DNA Polymerase (Cytiva), conditions: 0.5 µM template, 0.25 µM primer, 25 µM dNTP, 0.10 units/µL polymerase, 30 mM Tris-HCl, 7.5 mM $MgSO_4$, 60 °C.

2-Amino-DDP triphosphate was incorporated with KlenTaq using the conditions mentioned below with a total reaction volume of 80 µL. For every condition, samples were taken out after 1, 2, 5 and 15 min.

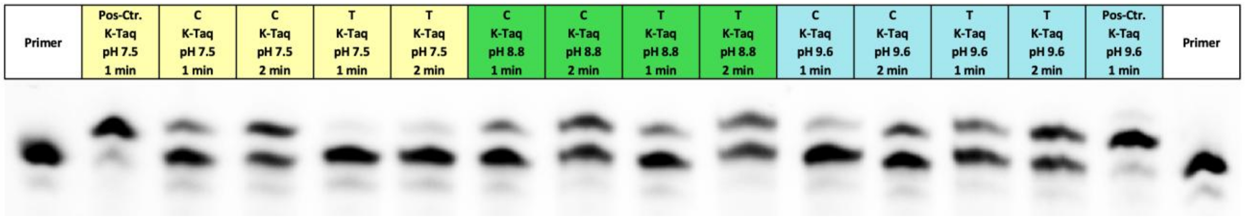

Figure S4: Urea-PAGE of the incorporation of 2-Amino-DDP triphosphate with KlenTaq. Conditions: 0.5 µM template, 0.25 µM primer, 25 µM dNTP, 0.05 units/µL polymerase, 25 mM Tris-HCl, 40 mM KCl, 5 mM $MgSO_4$, 60 °C.

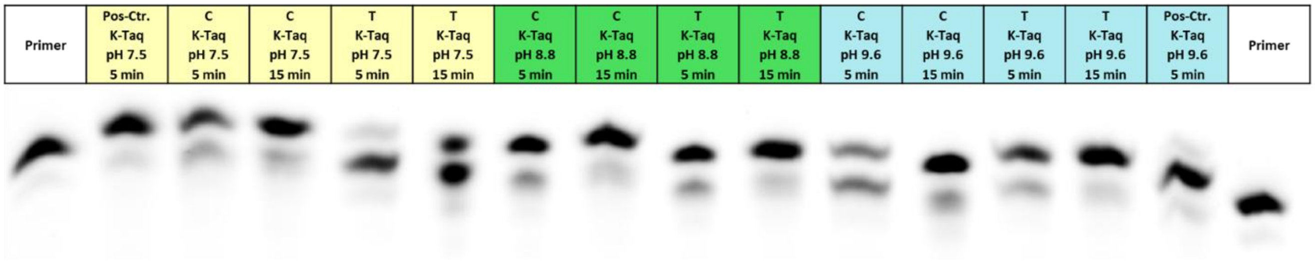

Figure S5: Urea-PAGE of the incorporation of 2-Amino-DDP triphosphate with KlenTaq. Conditions: 0.5 µM template, 0.25 µM primer, 25 µM dNTP, 0.05 units/µL polymerase, 25 mM Tris-HCl, 40 mM KCl, 5 mM $MgSO_4$, 60 °C.

UV/Vis and melting point measurements

The UV-Vis-absorption for melting point measurements and for the pK_a-determination was measured on an Agilent Cary 3500 Multicell Peltier UV-Vis spectrophotometer with Xenon Flashlamp Source. For the UV-Vis absorption, nucleoside **5** (1 mM) was dissolved in 50 mM sodium acetate buffer (for pH 5.0) or in 50 mM sodium phosphate buffers (for pH 6.5 to 9.5). The absorption at a wavelength of 200 to 600 nm was measured with a spectral bandwidth of 2.0 nm.

The 10mer DNA used for melting point measurements had the sequence TCAGXGTAAG. It was mixed with the corresponding template CTTACCCTGA or CTTACTCTGA at 3 µM oligonucleotide concentration in 10 mM phosphate buffer with pH from 6.0 to 9.5 and a total of 250 mM NaCl. Initially, the DNA was denatured at 80 °C and the strands were annealed by cooling to 10 °C for 10 min. The temperature was then raised to 60 °C at 2 °C/min, and the absorption was measured in 0.5 °C steps. After cooling down again to 10 °C, the measurements were repeated 3 times in total. The absorption at 260 nm and 420 nm was measured at each temperature and the difference was used to determine the melting points. The averaging time was set to 1.0 s and the spectral bandwidth to 2.0 nm.

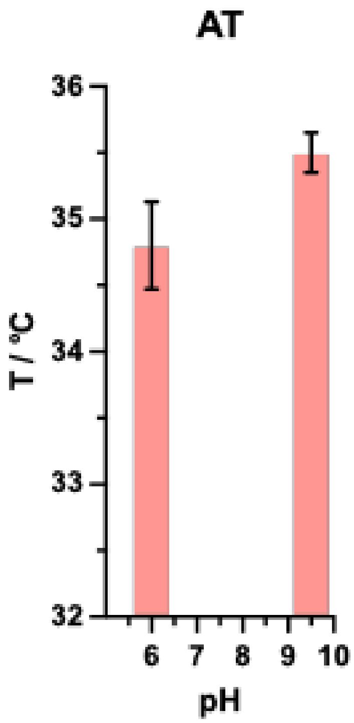

Figure S6: Melting points of the canonical AT-base pair 10mer had been measured at two different pH values by the UV absorption change at 260 nm.

Chemical synthesis

Compound 1b

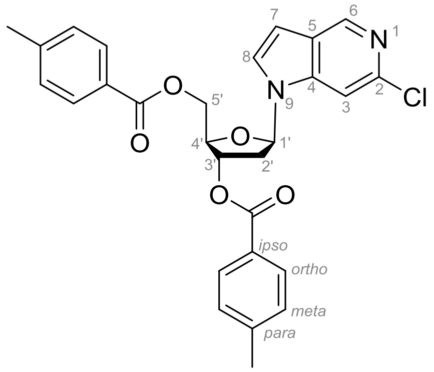

Chemical Formula: $C_{28}H_{25}ClN_2O_5$
Exact Mass: 504,1452
Molecular Weight: 504,9670

Freshly powdered KOH (3.67 g, 65.5 mmol, 5.00 eq.) was quickly mixed with dry THF (50 mL) under argon atmosphere. 6-Chloro-1H-pyrrolo[3,2-c]pyridine (2.00 g, 13.1 mmol, 1.00 eq.) and 1-Chloro-2-deoxy-3,5-di-*O*-toluoyl-ribose (5.61 g, 14.4 mmol, 1.10 eq.) were added and the resulting mixture was stirred for 30 min at room temperature. The brown mixture was filtered through Celite, washed with ethyl acetate and evaporated in vacuo. The crude product was purified via flash chromatography (20 to 50% EA in PE) to give **1b** (6.51 g, 12.9 mmol, 98%) as a yellow solid.

^{1}H NMR (500 MHz, $CDCl_3$) δ/ppm = 8.58 – 8.55 (m, 1H, H-6), 7.92 – 7.89 (m, 2H, $C^{5'OBz\text{-}ortho}$-$\underline{H}$), 7.85 – 7.81 (m, 2H, $C^{3'OBz\text{-}ortho}$-$\underline{H}$), 7.40 – 7.36 (m, 1H, H-3), 7.24 (d, *J* = 3.5 Hz, 1H, H-8), 7.23 – 7.15 (m, 4H, $C^{5'OBz\text{-}meta}$-$\underline{H}$ & $C^{3'OBz\text{-}meta}$-$\underline{H}$), 6.53 (dd, *J* = 3.5, 0.9 Hz, 1H, H-7), 6.30 (dd, *J* = 8.4, 5.6 Hz, 1H, H-1’), 5.63 (dt, *J* = 6.3, 2.5 Hz, 1H, H-3’), 4.59 – 4.51 (m, 3H, H-5’a & H-5’b & H-4’), 2.75 – 2.61 (m, 2H, H-2’a & H-2’b), 2.37 (s, 3H, $C^{5'OBz}$-$C\underline{H}_3$), 2.34 (s, 3H, $C^{3'OBz}$-$C\underline{H}_3$).

^{13}C NMR (126 MHz, $CDCl_3$) δ/ppm 166.3 ($\underline{C}$OOC-5’), 166.1 ($\underline{C}$OOC-3’), 144.8 ($C^{5'OBz\text{-}ipso}$), 144.4 ($C^{3'OBz\text{-}ipso}$), 143.4 (C-2), 142.9 (C-6), 142.9 (C-4), 129.9 ($C^{5'OBz\text{-}ortho}$), 129.8 ($C^{3'OBz\text{-}ortho}$), 129.5 ($C^{5'OBz\text{-}meta}$), 129.4 ($C^{3'OBz\text{-}meta}$), 126.8 ($C^{5'OBz\text{-}para}$), 126.5 ($C^{3'OBz\text{-}para}$), 126.3 (C-8), 125.4 (C-5), 105.2 (C-3), 103.3 (C-7), 85.6 (C-1’), 82.4 (C-4’), 74.9 (C-3’), 64.2 (C-5’), 38.4 (C-2’), 21.9 (Ar-$\underline{C}H_3$), 21.8 (Ar-$\underline{C}H_3$).

HRMS: Calculated for $[M+H]^+$ 505.1525, found: 505.1542.

Compound 3

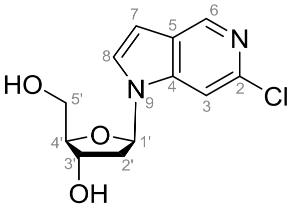

Chemical Formula: $C_{12}H_{13}ClN_2O_3$
Exact Mass: 268,0615
Molecular Weight: 268,6970

Cs_2CO_3 (620 mg, 1.90 mmol, 3.00 eq.) was added to a solution of **1b** (320 mg, 0.635 mmol, 1.00 eq.) in MeOH (50 mL) at room temperature and the mixture was stirred for 2 h. After removing the solvent in vacuo, water (50 mL) was added and it was extracted with EA (3 x 100 mL). The solvent was removed under reduced pressure and the residue was purified by flash column chromatography (1 to 10% MeOH in DCM) to give nucleoside **3** (173 mg, 0.571 mmol, 90%) as a white solid.

^{1}H NMR (700 MHz, Methanol-d_4) δ/ppm = 8.56 (s, 1H, H-6), 7.70 (s, 1H, H-3), 7.66 (d, *J* = 3.4 Hz, 1H, H-8), 6.70 (d, *J* = 3.4 Hz, 1H, H-7), 6.40 (dd, *J* = 6.8 Hz, 1H, H-1'), 4.51 (dt, *J* = 6.2, 3.6 Hz, 1H, H-3'), 4.00 – 3.95 (m, 1H, H-4'), 3.75 (dd, *J* = 12.1, 3.8 Hz, 1H, H-5'a), 3.70 (dd, *J* = 12.0, 4.3 Hz, 1H, H-5'b), 2.60 – 2.54 (m, 1H, H-2'a), 2.39 – 2.36 (m, 1H, H-2'b).

^{13}C NMR (176 MHz, Methanol-d_4) δ/ppm = 142.0 (C-2), 141.9 (C-4), 141.8 (C-6), 127.9 (C-8), 125.6 (C-5), 105.3 (C-3), 102.0 (C-7), 87.3 (C-4'), 85.2 (C-1'), 71.1 (C-3'), 61.8 (C-5'), 39.9 (C-2').

HRMS: Calculated for $[M+H]^+$ 269.0687, found: 269.0677.

Compound 3b

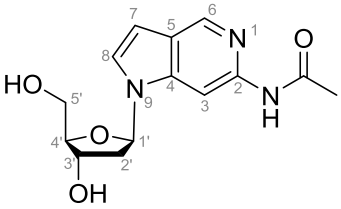

Chemical Formula: $C_{14}H_{17}N_3O_4$
Exact Mass: 291,1219
Molecular Weight: 291,3070

Nucleoside **3** (1.00 g, 3.72 mmol, 1.00 eq.) and acetamide (4.40 g, 74.4 mmol, 20.0 eq.) were mixed and heated to 130 °C. KOtBu (1.25 g, 11.2 mmol, 3.00 eq.) and tBu-BrettPhos Pd G3 (63.6 mg, 74.4 µmol, 0.02 eq.) were added under argon. The mixture was stirred at 130 °C for 16 h. After cooling down to room

temperature, the volatiles were evaporated in vacuo. The crude product was purified via flash chromatography on silica gel (MeOH 2 to 20% in DCM) to give the acetyl-protected nucleoside **3b** mixed with acetamide as orange crystals. It was directly used for the hydroxyl-group protection.

HRMS: Calculated for $[M+H]^+$ 292.1292, found: 292.1293.

Compound 4

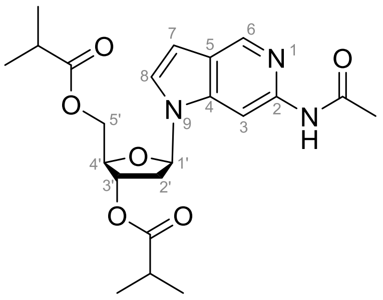

Chemical Formula: $C_{22}H_{29}N_3O_6$
Exact Mass: 431,2056
Molecular Weight: 431,4890

In a dry Schlenk tube, **3b** (600 mg, 2.06 mmol, 1.00 eq.) was dissolved in anhydrous pyridine (30.0 mL). Isobutyric anhydride (3.43 mL, 20.6 mmol, 10.0 eq.) and *N*-Methylimidazole (51 µL, 0.21 mmol, 0.10 eq.) were added and the resulting solution was stirred at room temperature under argon atmosphere for 30 min. The reaction mixture was quenched with methanol (30.0 mL), stirred for 10 minutes and evaporated in vacuo. The crude product was mixed with water (50 mL) and extracted with DCM (3x100 mL). The combined organic layers were washed with water (2x50 mL), dried over $MgSO_4$ and evaporated in vacuo. The product was purified via flash column chromatography (2 to 50% acetone in DCM) to give 732 mg (1.70 mmol, 82% over two steps) of the protected nucleoside **4** as a pale yellow solid.

^{1}H NMR (500 MHz, $CDCl_3$) δ/ppm = 9.65 (s, 1H, NH), 8.49 (s, 1H, H-6), 8.41 (s, 1H, H-3), 7.34 (d, *J* = 3.5 Hz, 1H, H-8), 6.64 (d, *J* = 3.5 Hz, 1H, H-7), 6.38 (dd, *J* = 8.2, 5.9 Hz, 1H, H-1'), 5.34 – 5.30 (m, 1HH-3'), 4.36 – 4.29 (m, 2H, H-5'a, H-5'b), 4.28 – 4.26 (m, 1H, H-4'), 2.70 – 2.63 (m, 1H, C^{iBu}-H), 2.62 – 2.58 (m, 1H, C^{iBu}-H), 2.57 – 2.49 (m, 2H, H-2'a, H-2'b), 2.25 (s, 3H, C^{Acetyl}-CH_3), 1.22 (dd, *J* = 6.9, 2.5 Hz, 6H, C^{iBu}-CH_3), 1.18 (dd, *J* = 6.9, 1.7 Hz, 6H, C^{iBu}-CH_3).

^{13}C NMR (126 MHz, $CDCl_3$) δ/ppm = 176.8 (C^{iBu}=O), 176.7 (C^{iBu}=O), 169.4 (C^{Acetyl}=O), 145.2 (C-2), 142.4 (C-4), 139.3 (C-6), 125.9 (C-8), 123.1 (C-5), 103.6 (C-7), 94.8 (C-3), 84.9 (C-1'), 82.2 (C-4'), 74.2 (C-3'), 64.0 (C-5'), 38.2 (C-2'), 34.1 (C^{iBu}H), 33.9 (C^{iBu}H), 24.8 ($C^{Acetyl}\underline{C}H_3$), 19.1 ($C^{iBu}\underline{C}H_3$), 19.1 ($C^{iBu}\underline{C}H_3$), 19.0 ($C^{iBu}\underline{C}H_3$), 19.0 ($C^{iBu}\underline{C}H_3$).

HRMS: Calculated for $[M+H]^+$ 432.2129, found: 432.2125.

Compound 5

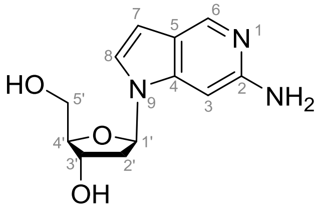

Chemical Formula: $C_{12}H_{15}N_3O_3$
Exact Mass: 249,1113
Molecular Weight: 249,2700

Compound **4b** (3.00 g, 6.95 mmol, 1.00 eq.) was stirred in a solution of 2 M NaOMe in MeOH at 80 °C for 18 h. The reaction mixture was evaporated in vacuo and fully dried via lyophilization, the crude product was purified via HPLC (5 to 20% ACN in water with 0.1% TFA) to give nucleoside **5** (1.13 g, 4.52 mmol, 65%) as white solid.

^{1}H NMR (600 MHz, Methanol-d_4) δ/ppm = 8.28 (s, 1H, H-6), 7.67 (d, *J* = 3.7 Hz, 1H, H-8), 6.93 (s, 1H, H-3), 6.67 (d, *J* = 3.7 Hz, 1H, H-7), 6.29 (dd, *J* = 6.8 Hz, 1H, H-1'), 4.50 (dt, *J* = 6.2, 3.1 Hz, 1H, H-3'), 4.00 (q, *J* = 3.8 Hz, 1H, H-4'), 3.78 – 3.64 (m, 2H, H-5'a, H-5'b), 2.55 (ddd, *J* = 13.7, 6.9 Hz, 1H, H-2'a), 2.38 (ddd, *J* = 13.6, 6.0, 3.2 Hz, 1H, H-2'b).

^{13}C NMR (151 MHz, Methanol-d_4) δ/ppm = 151.0 (C-2), 147.7 (C-4), 131.7 (C-8), 130.9 (C-6), 121.5 (C-5), 104.3 (C-7), 90.1 (C-3), 88.9 (C-4'), 86.5 (C-1'), 72.5 (C-3'), 63.2 (C-5'), 41.0 (C-2').

HRMS: Calculated for $[M+H]^+$ 250.1186, found: 250.1185.

Compound 5b

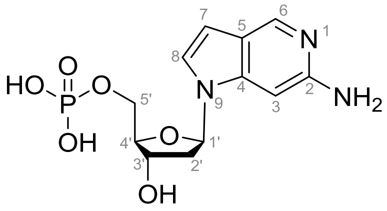

Chemical Formula: $C_{12}H_{16}N_3O_6P$
Exact Mass: 329,0777
Molecular Weight: 329,2488

In a dry Schlenk flask, compound 5 (50 mg, 0.138 mmol, 1.00 eq.) was dried under high vacuum, before trimethyl phosphate (3.0 mL) was added under an argon atmosphere. The mixture was cooled in an ice bath and $POCl_3$ (0.126 mL, 1.38 mmol, 10.0 eq.) was added dropwise. The reaction mixture was stirred at 0 °C for 20 min and at rt for 7 h, before a solution of 10% TEA in water (3 mL) was added and the mixture

was stirred for another 15 min at rt. The mixture was concentrated under reduced pressure and was purified via RP-HPLC, with 5 to 20% ACN in water containing 0.1% TFA to give the nucleoside monophosphate **5b** (25.0 mg, 56.4 µmol, 41%) as yellow oil.

^{1}H NMR (700 MHz, D_2O) δ/ppm = δ 8.16 (s, 1H, H-6), 7.53 (d, *J* = 3.7 Hz, 1H, H-8), 7.06 (s, 1H, H-3), 6.64 (d, *J* = 3.6 Hz, 1H, H-7), 6.31 (dd, *J* = 8.5, 6.0 Hz, 1H, H-1'), 4.69 (dt, *J* = 5.9, 2.7 Hz, 1H, H-3'), 4.23 (p, *J* = 2.8 Hz, 1H, H-4'), 4.10 (dd, *J* = 4.8, 3.2 Hz, 2H, H-5'a, H-5'b), 2.66 (ddd, *J* = 14.5, 8.6, 6.3 Hz, 1H, H-2'a), 2.39 (ddd, *J* = 14.1, 6.1, 2.6 Hz, 1H, H-2'b).

^{13}C NMR (176 MHz, D_2O) δ/ppm = 148.5 (C-4), 145.6 (C-2), 131.1 (C-8), 129.8 (C-6), 120.2 (C-5), 103.4 (C-7), 90.3 (C-3), 86.1 (C-1'), 85.3 (C-4'), 71.3 (C-3'), 64.9 (C-5'), 38.4 (C-2').

^{31}P NMR (283 MHz, D_2O) δ/ppm = 0.04.

HRMS: Calculated for $[M-H]^-$ 328.0704, found: 328.0706.

Compound 6

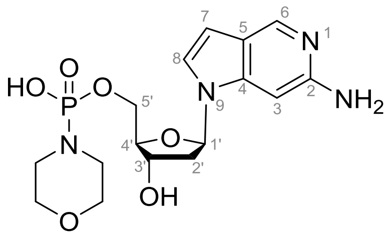

Chemical Formula: $C_{16}H_{23}N_4O_6P$
Exact Mass: 398,1355
Molecular Weight: 398,3558

In a dry Schlenk flask, compound **5b** (20.0 mg, 0.045 mmol, 1.00 eq.) was dissolved in dry DMSO (1.0 mL). Morpholine (39.3 mg, 0.451 mmol, 10.0 eq.) was added and the reaction mixture was stirred at room temperature for 5 min. Dipyridyl disulfide (29.8 mg, 0.135 mmol, 3.00 eq.) and triphenylphosphine (35.5 mg, 0.135 mmol, 3.00 eq.) were added and the reaction mixture was stirred for 2 h at room temperature. Water (10 mL) was added and the mixture was extracted with DCM (3 x 20 mL) to remove lipophilic components. The aqueous phase was dried by lyophilization and the crude product was purified via HPLC (5 to 20 % ACN in water) to give 11.0 mg (0.028 mmol, 61%) of the morpholidate **6** as yellow solid.

^{1}H NMR (600 MHz, D_2O) δ/ppm = 8.27 (s, 1H, H-6), 7.57 (d, *J* = 3.7 Hz, 1H, H-8), 7.14 (s, 1H, H-3), 6.72 (d, *J* = 3.4 Hz, 1H, H-7), 6.36 (dd, *J* = 7.7, 6.2 Hz, 1H, H-1'), 4.70 (dt, *J* = 6.5, 3.3 Hz, 1H, H-3'), 4.22 – 4.20 (m, 1H, H-4'), 3.97 – 3.95 (m, 2H, H-5'a, H-5'b), 3.62 – 3.54 (m, 4H, $O^{morpholidate}CH_2$), 2.98 – 2.93 (m,

4H, N^{morpholidate}CH$_2$), 2.76 (ddd, *J* = 14.1, 7.7, 6.4 Hz, 1H, H-2'a), 2.46 (ddd, *J* = 14.1, 6.2, 3.4 Hz, 1H, H-2'b).

^{13}C NMR (151 MHz, D$_2$O) δ/ppm = 148.7 (C-2), 146.0 (C-4), 130.9 (C-8), 130.1 (C-6), 120.2 (C-5), 103.5 (C-7), 90.4 (C-3), 85.9 (C-1'), 85.4 (C-4'), 71.1 (C-3'), 66.9 (O^{morpholidate}$\underline{C}$H$_2$), 64.4 (C-5'), 44.69 (N^{morpholidate}$\underline{C}$H$_2$), 38.2 (C-2').

^{31}P NMR (243 MHz, D$_2$O) δ/ppm = 7.37.

HRMS: Calculated for [M-H]$^-$ 397.1282, found: 397.1282.

Compound 7

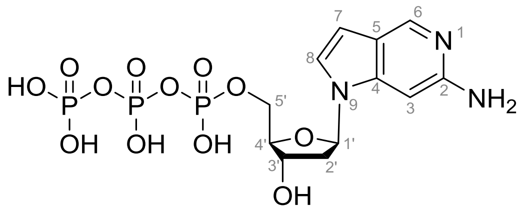

Chemical Formula: $C_{12}H_{18}N_3O_{12}P_3$
Exact Mass: 489,0103
Molecular Weight: 489,2063

Compound **6** (8.0 mg, 20.1 µmol, 1.00 eq.) was dried in a Schlenk flask. Anhydrous DMF (500 µL) was added and the solution was stirred at room temperature. Tributyl ammonium pyrophosphate (36.5 mg, 100.4 µmol, 5.00 eq) was added and the reaction mixture was stirred at rt for 24 h, before it was lyophilized. The crude product was purified using an HPLC with ion exchange column with Tris-buffer and a NaCl-solution with raising concentration (0 to 1.25 M NaCl). The resulting product fractions were desalted and purified using an HPLC with C18-column using a system of 100 mM TEAA-buffer (A) and a mixture of 20% 500 mM TEAA-buffer and 80% acetonitrile (B). The resulting triphosphate **7** was lyophilized and obtained as TEA-salt (2.84 mg, 3.58 µmol, 18%) and stored at -70 °C.

^{1}H NMR (600 MHz, D$_2$O) δ/ppm = 8.30 (d, *J* = 4.6 Hz, 1H, H-6), 7.55 (d, *J* = 3.7 Hz, 1H, H-8), 7.17 (s, 1H, H-3), 6.70 (d, *J* = 3.6 Hz, 1H, H-7), 6.36 (dd, *J* = 8.3, 6.2 Hz, 1H, H-1'), 4.85 – 4.83 (m, 1H, H-3'), 4.44 – 4.40 (m, 1H, H-4'), 4.31 – 4.27 (m, 1H, H-5'a), 4.23 – 4.19 (m, 1H, H-5'b), 2.75 (dt, *J* = 14.5, 7.5 Hz, 1H, H-2'a), 2.41 (dt, *J* = 14.1, 4.0 Hz, 2H, H-2'b).

^{13}C NMR (151 MHz, D$_2$O) δ/ppm = 149.6 (C-4), 145.3 (C-2), 132.2 (C-6), 130.5 (C-8), 120.7 (C-5), 103.3 (C-7), 90.7 (C-3), 86.2 (C-1'), 85.6 (C-4'), 70.8 (C-3'), 65.0 (C-5'), 38.4 (C-2').

^{31}P NMR (243 MHz, D$_2$O) δ/ppm = -7.38 – -7.56 (m, 1P, γ-P), -11.07 (dd, *J* = 66.0, 19.9 Hz, 1P, α-P), -22.15 – -22.42 (m, 1P, β-P).

HRMS: Calculated for $[M\text{-}H]^-$ 488.0031, found: 488.0019.

***N,N*-dibutyl formamide dimethyl acetal**

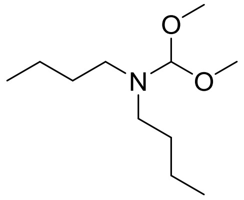

Chemical Formula: $C_{11}H_{25}NO_2$
Molecular Weight: 203,3260

N,*N*-Dibutyl formamide (28.9 mL, 159 mmol, 1.00 eq.) and dimethyl sulfate (15.1 mL, 159 mmol, 1.00 eq.) were mixed and stirred at 90 °C for 3 d. The resulting brown reaction mixture was then cooled to room temperature, before diethyl ether (20 mL) was slowly added over 10 min while stirring. The upper, ethereal layer was removed with a cannula, and diethyl ether (20 mL) was added to the residue. The upper, ethereal layer was removed again. The remaining brown mixture was slowly poured into a rapidly stirring, cold (0 °C) solution of 5.4 M sodium methoxide in methanol (88.3 mL, 477 mmol, 3.00 eq.) and was stirred for 16 h at room temperature, before it was extracted with cyclohexane (5 × 100 mL). The combined organic extracts were dried over sodium sulfate and concentrated in a low vacuum to a yellow liquid. The mixture was distilled under high vacuum with a slowly raising bath temperature (final temperature: 130 °C) to give 15.9 g (78.0 mmol, 49%) of *N*,*N*-dibutyl formamide dimethyl acetal as a clear, colorless liquid (bp 62 °C at 1.5 mbar). The collected fraction contained 11% *N*,*N*-dibutyl formamide (determined by NMR).

^{1}H NMR (500 MHz, $CDCl_3$) δ/ppm = 4.52 (s, 1H, $NC\underline{H}(OCH_3)_2$), 3.31 (s, 6H, OCH_3), 2.63 – 2.57 (m, 4H, $N(C\underline{H}_2CH_2CH_2CH_3)_2$), 1.45 – 1.38 (m, 4H, $N(CH_2C\underline{H}_2CH_2CH_3)_2$), 1.34 – 1.26 (m, 4H, $N(CH_2CH_2C\underline{H}_2CH_3)_2$), 0.90 (t, *J* = 7.3 Hz, 6H, $N(CH_2CH_2CH_2C\underline{H}_3)_2$).

^{13}C NMR (126 MHz, $CDCl_3$) δ/ppm = 112.8 ($N\underline{C}H(OCH_3)_2$), 54.1 (OCH_3), 47.3 ($N(\underline{C}H_2CH_2CH_2CH_3)_2$), 31.2 ($N(CH_2\underline{C}H_2CH_2CH_3)_2$), 20.7($N(CH_2CH_2\underline{C}H_2CH_3)_2$), 14.2 ($N(CH_2CH_2CH_2\underline{C}H_3)_2$).

The product is not stable under LC-MS conditions; therefore, no exact mass could be determined.

Compound 9

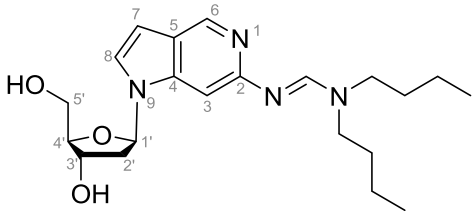

Chemical Formula: $C_{21}H_{32}N_4O_3$
Exact Mass: 388,2474
Molecular Weight: 388,5120

Nucleoside **5** (160 mg, 0.64 mmol, 1.00 eq.) was dissolved in THF (5 mL). *N*,*N*-Dibutyl formamide dimethyl acetal (0.35 mL, 1.93 mmol, 3.00 eq.) was added and the mixture was stirred at rt for 3 days. The reaction mixture was concentrated under reduced pressure. The crude product was purified via HPLC to give 240 mg (0.62 mmol, 95%) of **9**.

^{1}H NMR (700 MHz, Methanol-d_4) δ/ppm = 8.44 (s, 1H, H-6), 8.44 (s, 1H, N=C$\underline{H}$N), 7.66 (d, J = 3.5 Hz, 1H, H-8), 7.51 (s, 1H, H-3), 6.67 (d, J = 3.5 Hz, 1H, H-7), 6.36 (dd, J = 7.5, 6.2 Hz, 1H, H-1'), 4.46 (dt, J = 6.2, 3.1 Hz, 1H, H-3'), 3.95 (q, J = 3.8 Hz, 1H, H-4'), 3.73 – 3.65 (m, 2H, H-5'), 3.52 (t, J = 7.8 Hz, 2H, (N(C$\underline{H}_2$CH$_2$CH$_2$CH$_3$)$_2$)), 3.44 (t, J = 7.4 Hz, 2H, (N(C$\underline{H}_2$CH$_2$CH$_2$CH$_3$)$_2$)), 2.50 (ddd, J = 13.8, 7.5, 6.3 Hz, 1H, H-2'a), 2.34 (ddd, J = 13.6, 6.0, 3.2 Hz, 1H, H-2'b), 1.64 – 1.60 (m, 4H, (N(CH$_2$C$\underline{H}_2$CH$_2$CH$_3$)$_2$)), 1.38 – 1.30 (m, 4H, (N(CH$_2$CH$_2$C$\underline{H}_2$CH$_3$)$_2$)), 0.95 – 0.88 (m, 6H, (N(CH$_2$CH$_2$CH$_2$C$\underline{H}_3$)$_2$)).

^{13}C NMR (176 MHz, Methanol-d_4) δ/ppm = 156.7 (N$\underline{C}$H=N), 152.1 (C-2), 145.8 (C-4), 135.9 (C-6), 131.1 (C-8), 124.1 (C-5), 104.3 (C-7), 94.7 (C-3), 88.9 (C-4'), 86.8 (C-1'), 72.5 (C-3'), 63.2 (C-5'), 53.7 (N($\underline{C}$H$_2$CH$_2$CH$_2$CH$_3$)$_2$), 47.1 (N($\underline{C}$H$_2$CH$_2$CH$_2$CH$_3$)$_2$), 41.3 (C-2'), 29.2 (N(CH$_2$$\underline{C}H_2CH_2CH_3$)$_2$), 20.7 (N(CH$_2CH_2$$\underline{C}H_2CH_3$)$_2$), 14.0 (N(CH$_2CH_2CH_2$$\underline{C}H_3$)$_2$).

HRMS: Calculated for $[M+H]^+$ 389.2547, found: 389.2537.

Compound 10

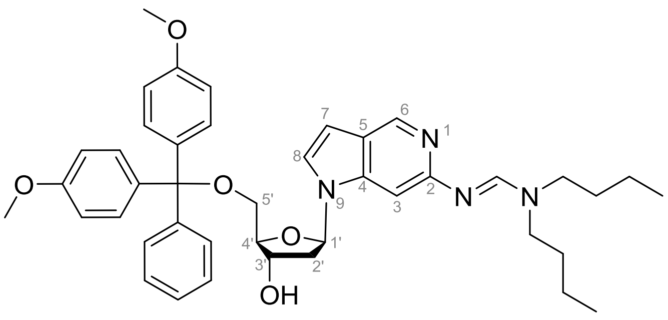

Chemical Formula: $C_{42}H_{50}N_4O_5$
Exact Mass: 690,3781
Molecular Weight: 690,8850

In a dry Schlenk tube, **9** (200 mg, 0.515 mmol, 1.00 eq.) and 4,4´-dimethoxytritylchloride (523 mg, 1.54 mmol, 3.00 eq.) were dried under high vacuum, before anhydrous THF (15.0 mL) and DIPEA (0.44 mL, 2.57 mmol, 5.00 eq.) were added at rt. The resulting yellow mixture was stirred at rt for 16 h, before MeOH (5 mL) was added and stirring was continued for another 5 min. The reaction mixture was evaporated to dryness under reduced pressure. The crude product was purified via flash column chromatography (30% to 70% acetone in DCM with 0.1% TEA) to give 92.0 mg (0.133 mmol, 26%) of the DMT-protected product **10** as yellow solid.

^{1}H NMR (600 MHz, Methanol-d_4) δ/ppm = 8.45 (d, *J* = 0.9 Hz, 1H, H-6), 8.20 (s, 1H, N=CHN), 7.37 – 7.36 (m, 1H, C^{DMT}-H^{para}), 7.36 – 7.35 (m, 1H, $C^{Ar}H$), 7.33 (d, *J* = 3.5 Hz, 1H, H-8), 7.24 – 7.21 (m, 4H, C^{DMT}-H^{meta}), 7.18 – 7.09 (m, 4 H, $C^{Phenyl}H$), 6.73 – 6.70 (m, 4H, C^{DMT}-H^{ortho}), 6.50 (d, *J* = 3.5 Hz, 1H, H-7), 6.37 (dd, *J* = 6.4 Hz, 1H, H-1’), 4.53 (dt, *J* = 6.4, 4.1 Hz, 1H, H-3’), 4.06 (dt, *J* = 4.9, 3.6 Hz, 1H, H-4’), 3.70 (s, 6H, OCH_3), 3.32 – 3.25 (m, 4H, $N(C\underline{H}_2CH_2CH_2CH_3)_2$), 2.65 (ddd, *J* = 13.3, 6.5 Hz, 1H, H-2’a), 2.40 (ddd, *J* = 13.5, 6.2 Hz, 1H, H-2’b), 1.68 – 1.56 (m, 4H, $N(CH_2C\underline{H}_2CH_2CH_3)_2$), 1.34 – 1.26 (m, 4H, $N(CH_2CH_2C\underline{H}_2CH_3)_2$), 0.96 (tt, *J* = 14.9, 7.4 Hz, 6H, $N(CH_2CH_2CH_2C\underline{H}_3)_2$).

^{13}C NMR (151 MHz, Methanol-d_4) δ/ppm = 160.0 ($\underline{C}^{DMT}$-O-CH_3), 156.9 (C-2), 156.8 (N=$\underline{C}$HN), 146.3 (C-4), 141.1 (C-6), 137.1 (C^{DMT}-H^{para}), 131.3 (C^{DMT}-H^{meta}), 129.3 (C^{DMT}-H^{para}), 128.7 (C^{DMT}-H^{meta}), 127.4 (C-8), 124.4 (C-5), 114.0 (C^{DMT}-H^{meta}), 103.3 (C-7), 97.5 (C-3), 87.5 (C^{DMT}-O-5’), 87.1 (C-4’), 86.1 (C-1’), 72.6 (C-3’), 65.2 (C-5’), 55.7 (O-CH_3), 52.9 ($N(\underline{C}H_2CH_2CH_2CH_3)_2$), 40.8 (C-2’), 32.3 ($N(CH_2\underline{C}H_2CH_2CH_3)_2$), 30.3 $N(CH_2\underline{C}H_2CH_2CH_3)_2$), 21.2 ($N(CH_2CH_2\underline{C}H_2CH_3)_2$), 20.8 ($N(CH_2CH_2\underline{C}H_2CH_3)_2$), 14.3 ($N(CH_2CH_2CH_2\underline{C}H_3)_2$), 14.1 ($N(CH_2CH_2CH_2\underline{C}H_3)_2$).

HRMS: Calculated for $[M+H]^+$ 691.3854, found: 691.3827.

Compound 12

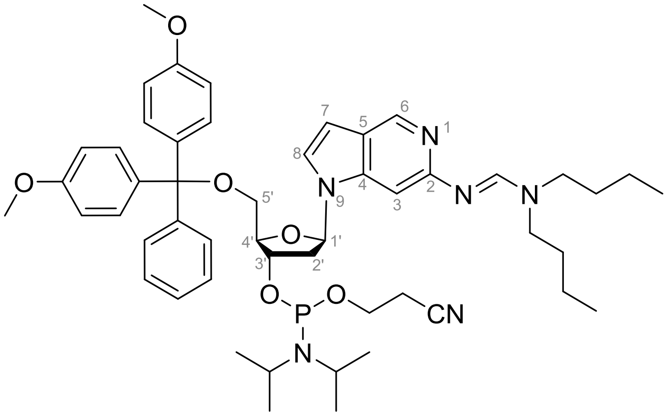

Chemical Formula: $C_{51}H_{67}N_6O_6P$
Exact Mass: 890,4860
Molecular Weight: 891,1068

After drying in high vacuum, **10** (92.0 mg, 0.133 mmol, 1.00 eq.) was dissolved in dry DCM (1.0 mL) containing DIPEA (69.8 µL, 0.399 mmol, 3.00 eq.). 2-Cyanoethyl-*N*,*N*-diisopropylphosphoramidochloridite (Compound 12, 59.5 µL, 0.266 mmol, 2.00 eq.) was added to the solution under argon and the reaction mixture was stirred for 30 min at rt. The mixture was concentrated in vacuo and purified via flash column chromatography (20 to 70% EA in CH) to give **12** (104 mg, 0.117 mmol, 88%).

^1H NMR (600 MHz, Acetonitrile-d_3) δ/ppm = 8.54 (s, 1H, N=CHN), 8.46 (d, *J* = 0.9 Hz, 1H, H-6), 7.39 – 7.36 (m, 2H, $H^{Ar,DMT}$), 7.27 – 7.21 (m, 7H, H-8, $H^{Ar,DMT}$), 7.21 – 7.17 (m, 1H, $H^{Ar,DMT}$), 6.90 – 6.89 (m, 1H, H-3), 6.78 – 6.75 (m, 4H, $H^{ortho,DMT}$), 6.50 (dd, *J* = 3.4, 0.9 Hz, 1H, H-7), 6.33 (t, *J* = 6.3 Hz, 1H, H-1'), 4.72 (ddt, *J* = 11.0, 6.6, 4.5 Hz, 1H, H-3'), 4.13 (tdd, *J* = 4.6, 3.3, 0.9 Hz, 1H, H-4'), 3.74 (s, 3H, OCH_3), 3.73 (s, 3H, OCH_3), 3.71 – 3.57 (m, 3H, $POC\underline{H}_2CH_2CN$, $N^{iPr}(C\underline{H}(CH_3)_2)_2$), 3.48 (t, *J* = 7.6 Hz, 2H, $N(C\underline{H}_2CH_2CH_2CH_3)_2$), 3.33 – 3.29 (m, 2H, $N(C\underline{H}_2CH_2CH_2CH_3)_2$), 3.28 (dd, *J* = 10.5, 3.4 Hz, 1H, H-5'a), 3.13 (dd, *J* = 10.5, 4.7 Hz, 1H, H-5'b), 2.72 – 2.65 (m, 1H, H-2'a), 2.55 – 2.48 (m, 3H, H-2'b, $POCH_2C\underline{H}_2CN$), 1.67 – 1.55 (m, 4H, $N(CH_2C\underline{H}_2CH_2CH_3)_2$), 1.39 – 1.30 (m, 4H, $N(CH_2CH_2C\underline{H}_2CH_3)_2$), 1.20 – 1.15 (m, 12H, $N^{iPr}(CH(C\underline{H}_3)_2)_2$), 0.95 (q, *J* = 8.9, 8.5 Hz, 6H, $N(CH_2CH_2CH_2C\underline{H}_3)_2$).

^{13}C NMR (151 MHz, Acetonitrile-d_3) δ/ppm = 159.6 (C^{DMT}-O-CH_3), 157.7 (C-2), 155.2 (N=CHN), 146.1 ($C^{Ar,DMT}$), 144.0 (C-4), 142.2 (C-6), 136.8 ($C^{Ar,DMT,meta}$), 131.0 ($C^{Ar,DMT,meta}$), 129.0 ($C^{Ar,DMT,meta}$), 128.8 (C-8), 127.7 ($C^{Ar,DMT}$), 125.6 ($C^{Ar,DMT}$), 123.6 (C-5), 119.4 ($POCH_2CH_2\underline{C}N$), 114.0 ($C^{Ar,DMT,ortho}$), 102.9 (C-7), 98.0 (C-3), 86.9 (C^{DMT}-O-5'), 85.6 (C-1'), 85.5 (C-4'), 73.8 (C-3'), 64.3 (C-5'), 59.5 ($PO\underline{C}H_2CH_2CN$), 55.9 (2 C, OCH_3), 51.9 ($N^{iPr}(\underline{C}H(CH_3)_2)_2$), 51.9 ($N(\underline{C}H_2CH_2CH_2CH_3)_2$), 45.5 ($N(\underline{C}H_2CH_2CH_2CH_3)_2$), 39.4 (C-2'), 32.1 ($N(CH_2\underline{C}H_2CH_2CH_3)_2$), 30.1 ($N(CH_2\underline{C}H_2CH_2CH_3)_2$), 24.9 (4 C, $N^{iPr}(CH(\underline{C}H_3)_2)_2$), 21.0 ($POCH_2\underline{C}H_2CN$), 21.0 ($N(CH_2CH_2\underline{C}H_2CH_3)_2$), 20.9 ($N(CH_2CH_2\underline{C}H_2CH_3)_2$), 14.3 ($N(CH_2CH_2CH_2\underline{C}H_3)_2$), 14.1 ($N(CH_2CH_2CH_2\underline{C}H_3)_2$).

The product oxidizes under LC-MS conditions to the structure depicted below.

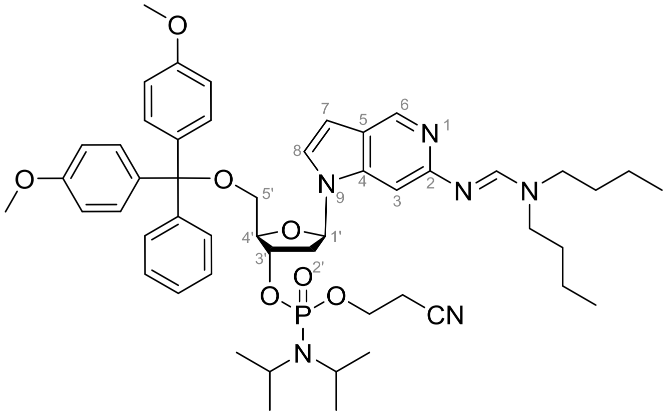

Chemical Formula: $C_{51}H_{67}N_6O_7P$
Exact Mass: 906,4809
Molecular Weight: 907,1058

HRMS: Calculated for $[M+H]^+$ 907.4882, found: 907.4886.

NMR- and MS-spectra

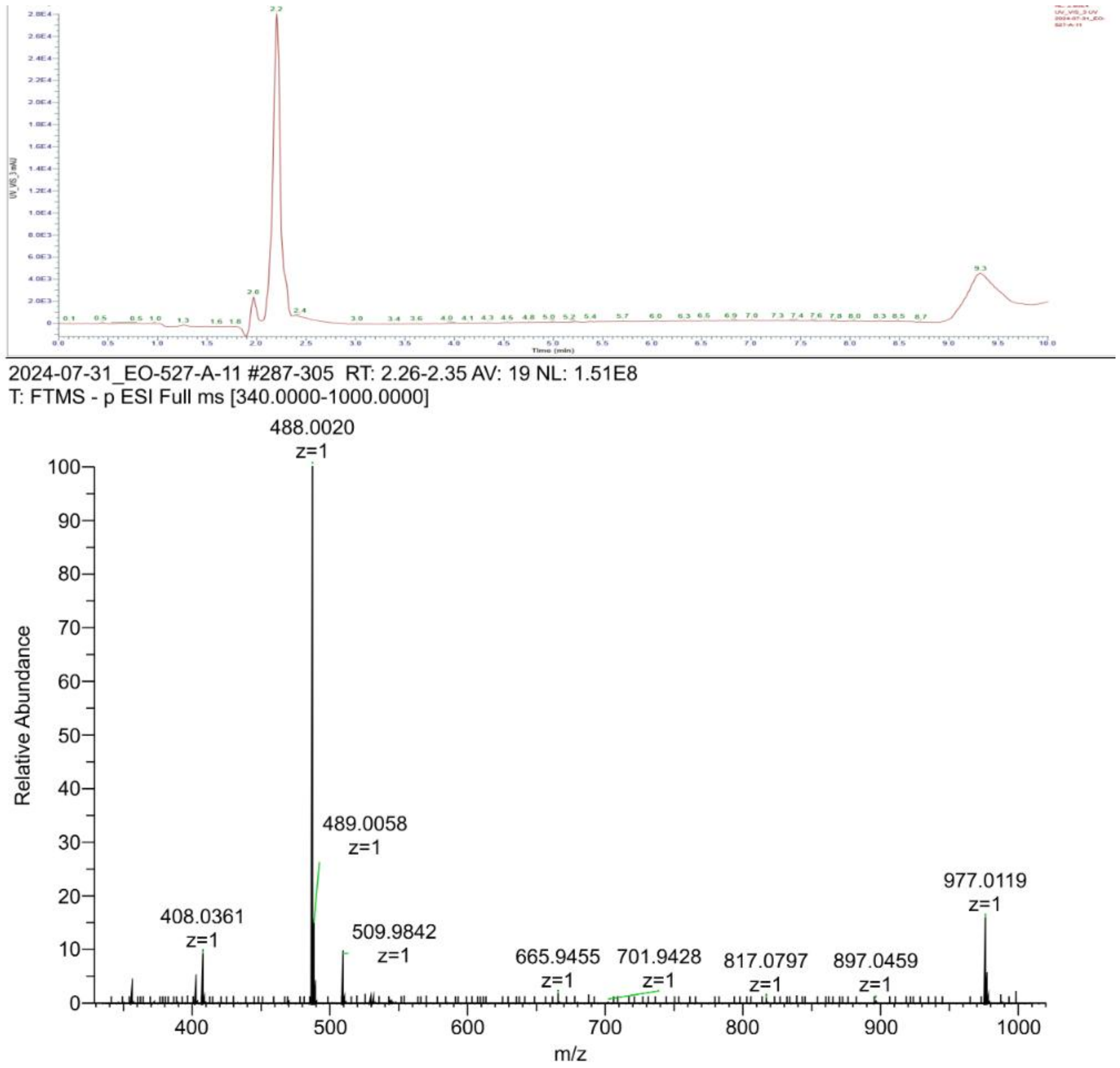

Figure S7: HRMS-Spectrum of compound 7.

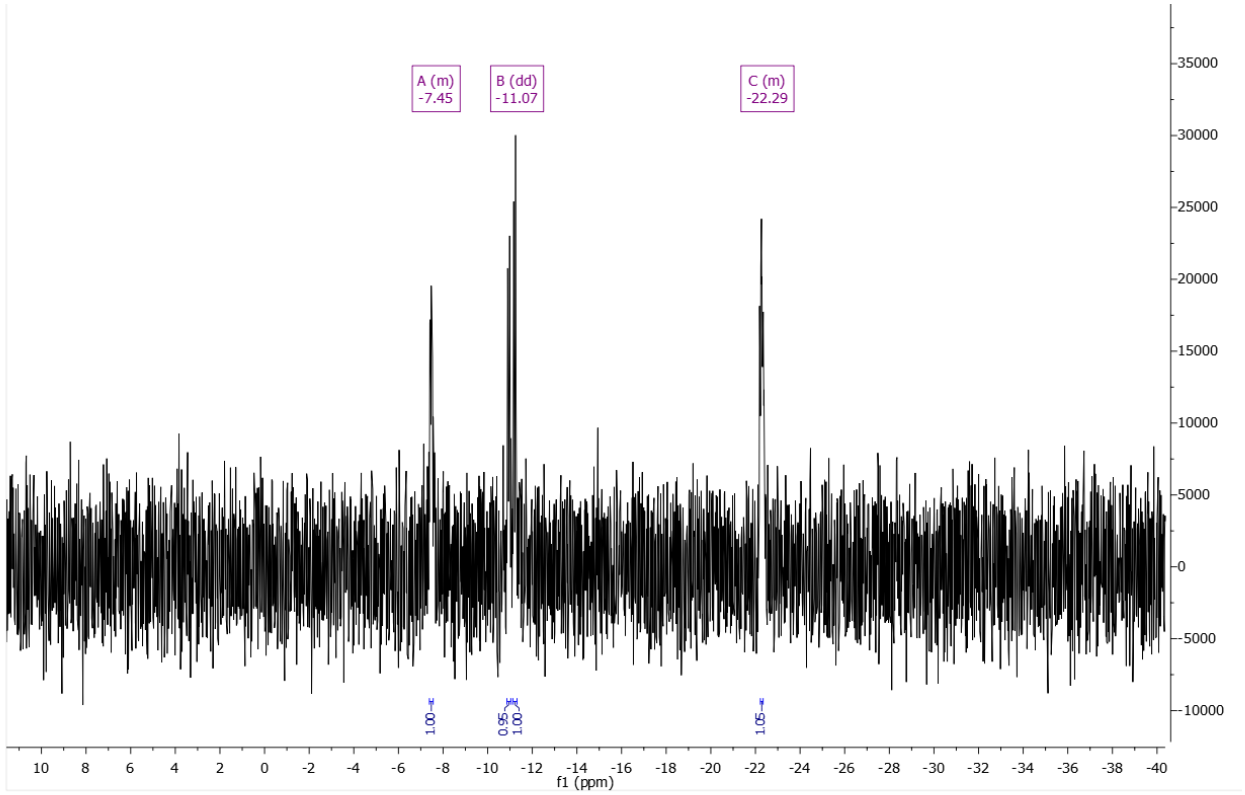

Figure S8: ^{31}P-NMR-Spectrum of compound 7.

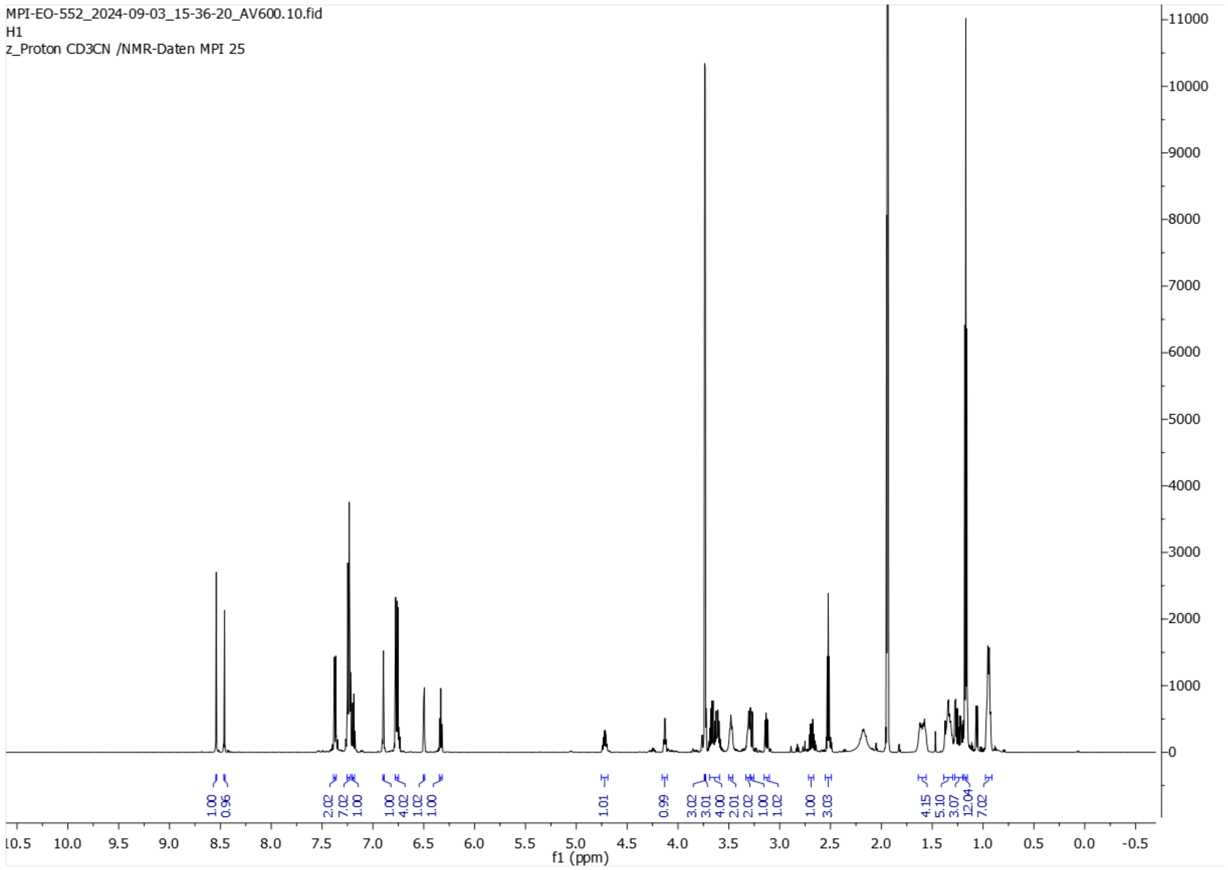

Figure S9: ^{1}H-NMR-Spectrum of compound 12.

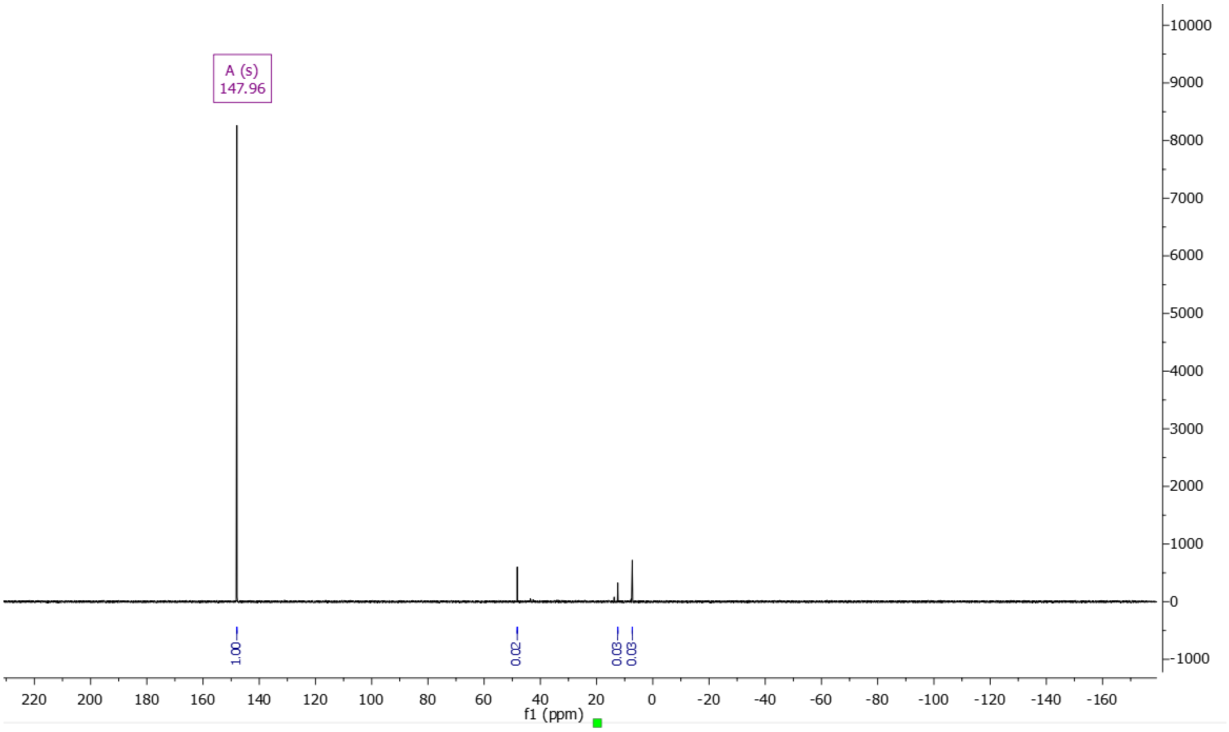

Figure S10: ^{31}P-NMR-Spectrum of compound 12.

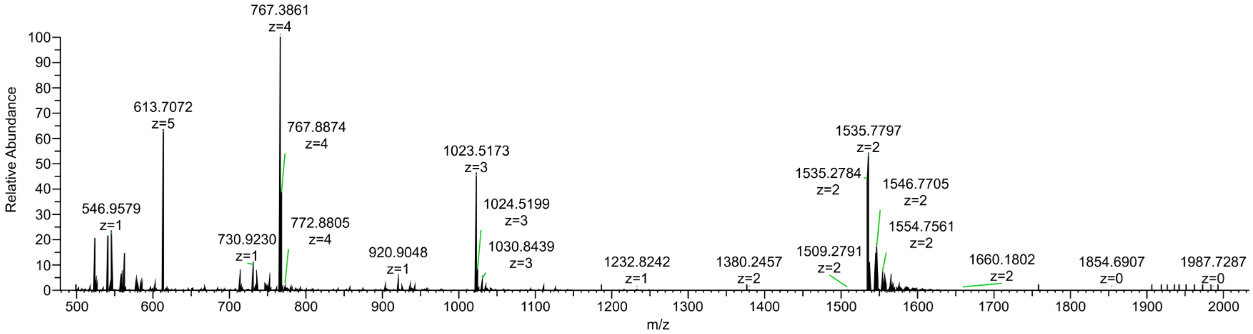

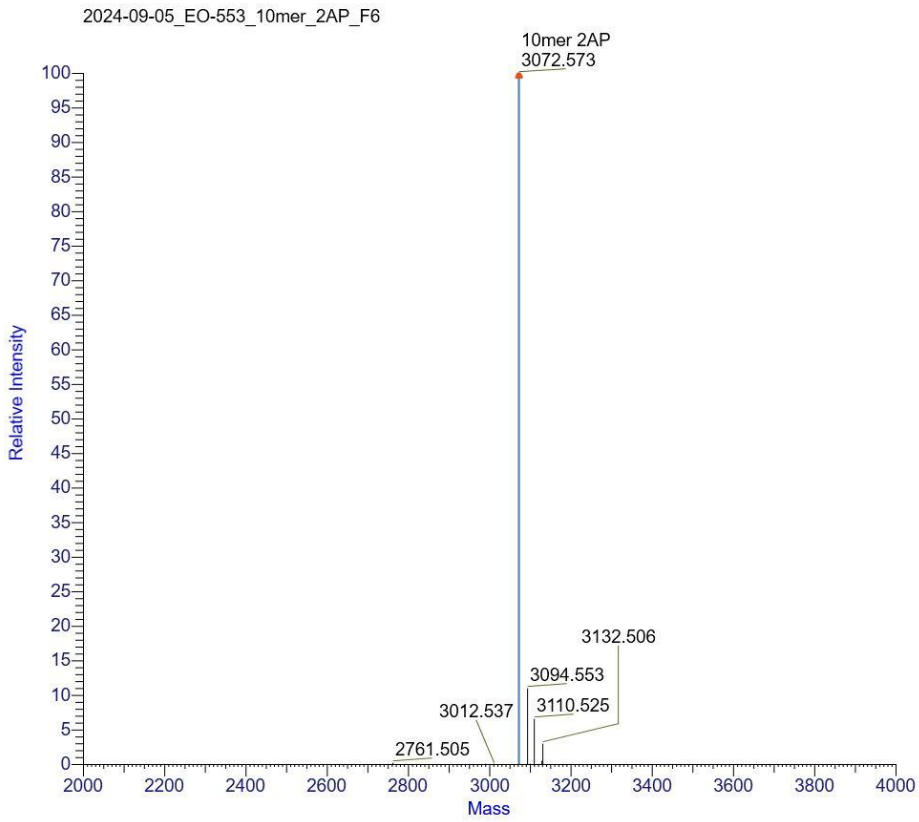

Figure S11: HRMS-Spectrum (top) and deconvoluted mass (bottom) of DNA-strand TCAGXGTAAG (with X=2-Amino-DDP). The calculated mass is 3072.580, the deconvoluted mass is 3072.573.

Primer extension on a 49-mer template with a 30-mer primer

Table 1: Calculated and deconvoluted masses of the oligonucleotides

Oligonucleotide	Calculated mass	Deconvoluted mass
30-mer primer	9676.731	Not detected after extension
31-mer primer (+X)	9985.782	Not detected after extension

32-mer primer (+XX)	10296.849	10296.861
49-mer Template	14981.430	Not detected after extension
50-mer Template (+A)	15292.472	15292.432
49-mer primer (fully extended)	15591.7284	Not detected after extension
50-mer primer (extended + A-Tail)	15904.786	15904.762

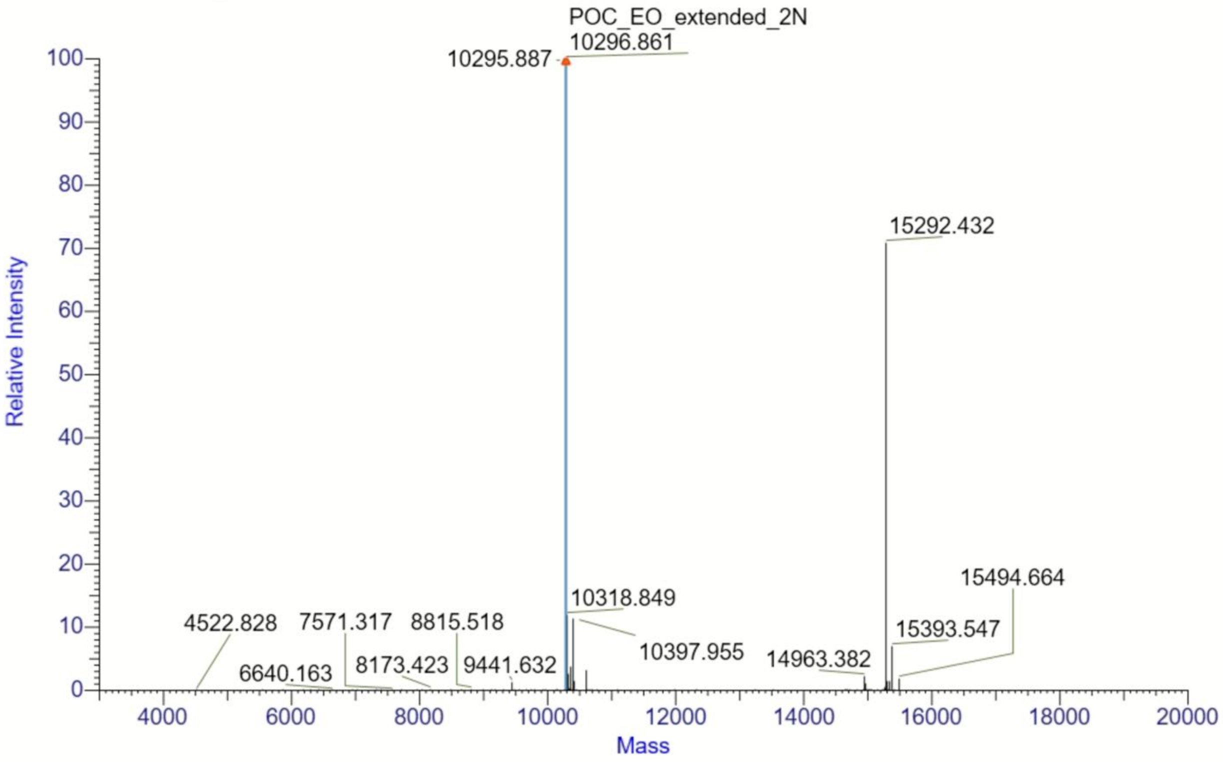

*Figure S12: HRMS-Spectrum of the mixture of elongated (Btn)-primer CGG GCG GAC CAG AAC CCT TGA GCA CAG AAA **XX** (with X=2-Amino-DDP) and the template (incl. A-tail). The calculated mass is 10296.849, the deconvoluted mass is 10296.861. The calculated mass of the template with single A-tailing is 15292.472, the deconvoluted mass is 15292.432.*

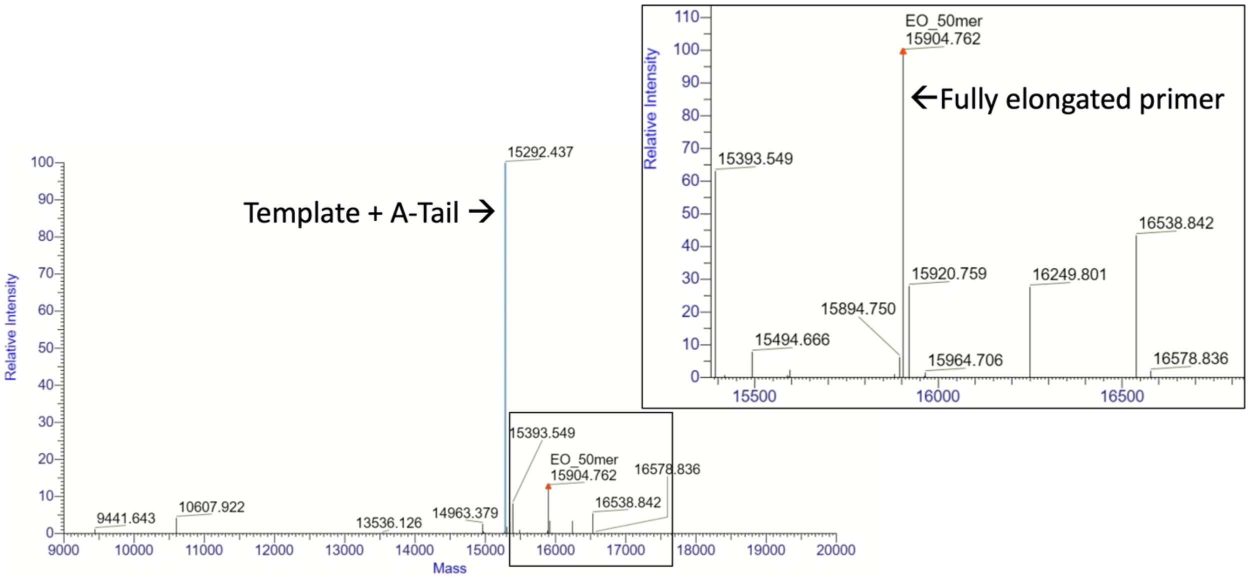

Figure S13: Deconvoluted HRMS-Spectrum of the elongated 50-mer (Btn)-primer CGG GCG GAC CAG AAC CCT TGA GCA CAG AAA ***XX****C GTC GAG TTA GCC GAA GA (with X=2-Amino-DDP). The calculated mass is 15904.786, the deconvoluted mass we detected is 15904.762.*

Unedited gel images

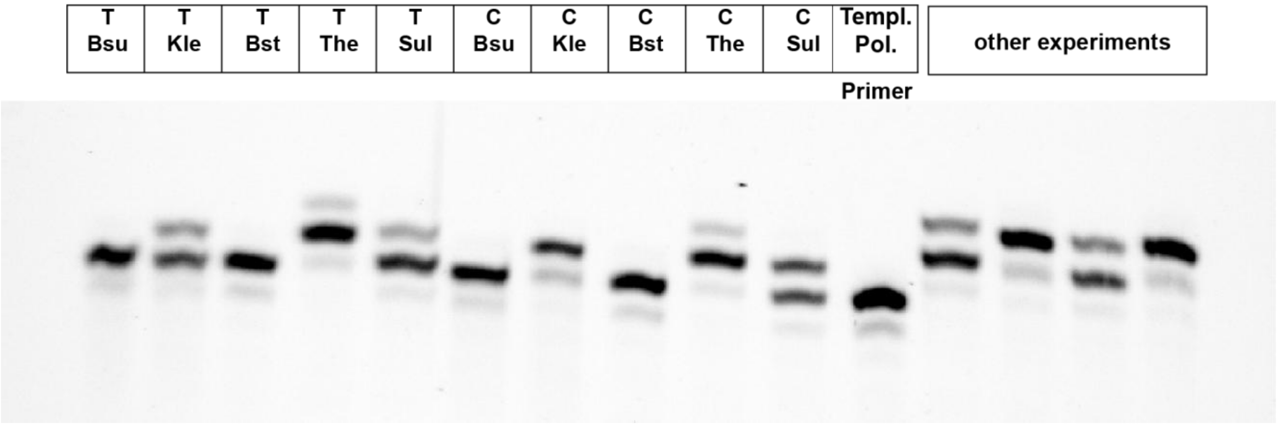

Figure S14: Unedited image of Figure 2c. Urea-PAGE of the enzymatic incorporation of 2-Amino-DDP with different polymerases. Conditions: 0.5 µM template, 0.25 µM primer, 100 µM dNTP, 0.05 units/µL polymerase, 10 min. Temp: 60 °C for Bst, Kle and Sul, 37 °C for Bsu and Kle. Bsu (Bsu DNA Polymerase, Large Fragment), Kle (Klenow-Fragment), Bst (Bst DNA Polymerase, Large Fragment), The (Therminator™ DNA Polymerase), Sul (Sulfolobus DNA Polymerase IV).

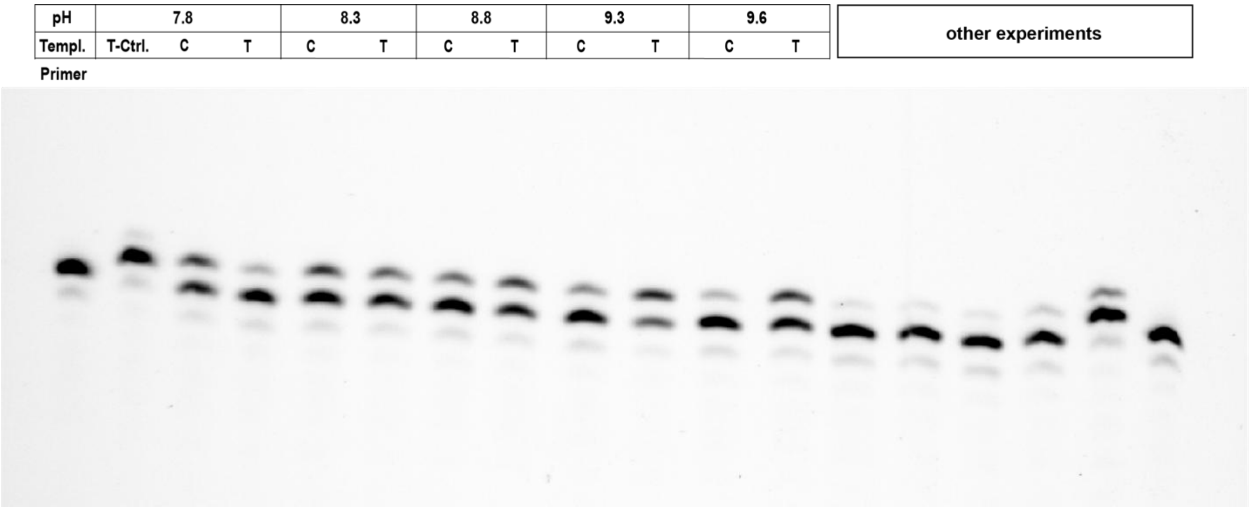

Figure S15: Unedited image of Figure 2d. Urea-PAGE of the enzymatic and pH-dependent incorporation of 2-Amino-DDP with Thermo Sequenase™ DNA Polymerase (Cytiva), conditions: 0.5 µM template, 0.25 µM primer, 25 µM dNTP, 0.05 units/µL polymerase, 30 mM Tris-HCl, 7.5 mM $MgSO_4$, 60 °C, 10 min.

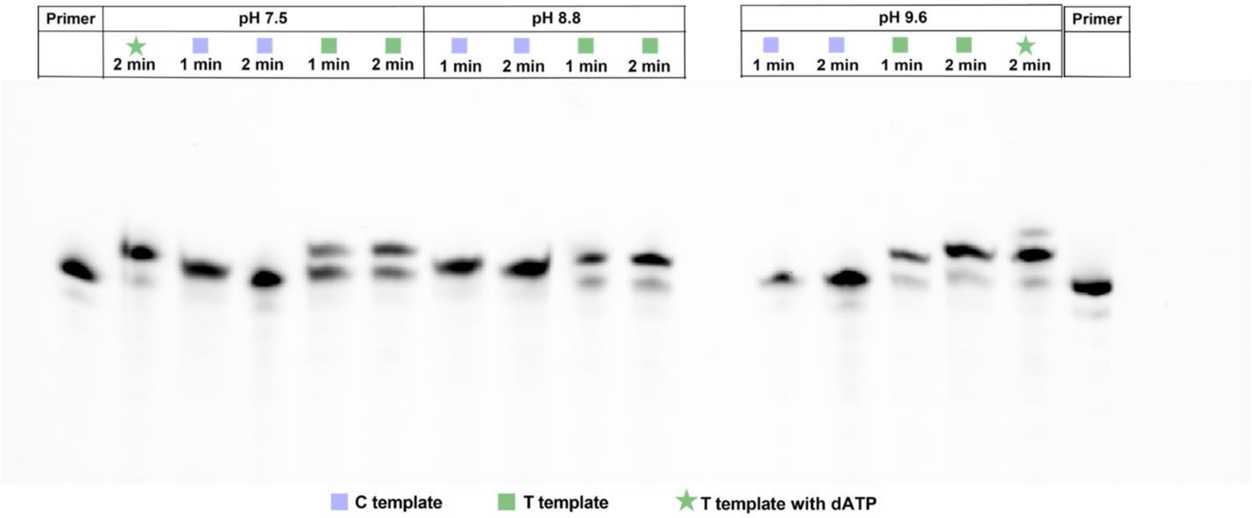

Figure S16: Unedited image of Figure S1. Urea-PAGE of the incorporation of 2-AP with KlenTaq. Conditions: 0.5 µM template, 0.25 µM primer, 25 µM dNTP, 0.05 units/µL polymerase, 25 mM Tris-HCl, 40 mM KCl, 5 mM $MgSO_4$, 60 °C. The experiments where dATP was incorporated opposite a T template were conducted as positive control.

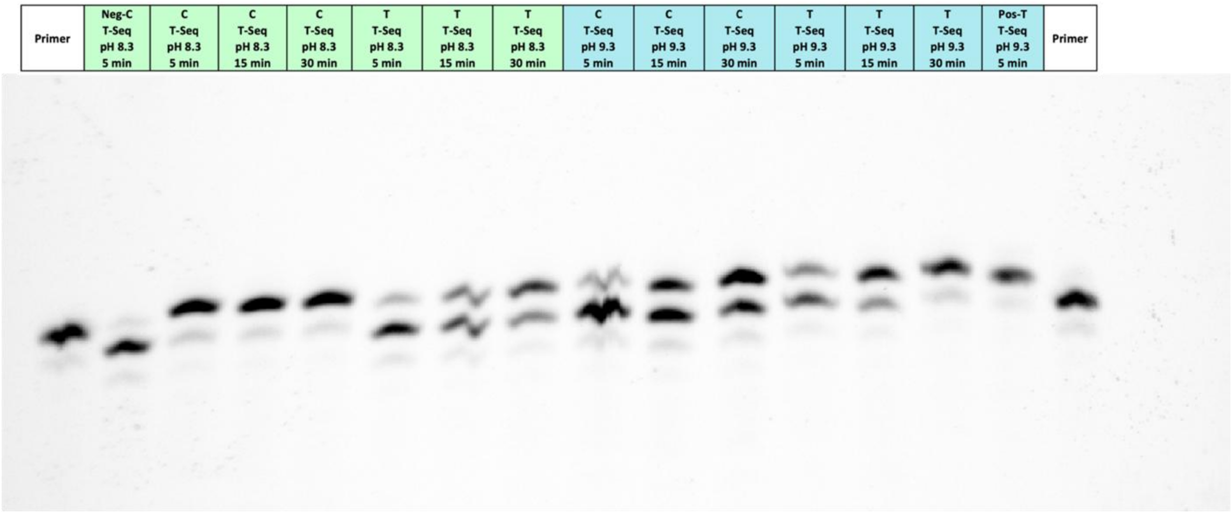

Figure S17: Unedited image of Figure S2. Urea-PAGE of the incorporation of 2-Amino-DDP triphosphate with Thermo Sequenase™ DNA Polymerase (Cytiva), conditions: 0.5 µM template, 0.25 µM primer, 25 µM dNTP, 0.10 units/µL polymerase, 30 mM Tris-HCl, 7.5 mM $MgSO_4$, 60 °C. Neg-C (negative control): no dNTP added. Pos-C (positive control): 25 µM dATP as only dNTP.

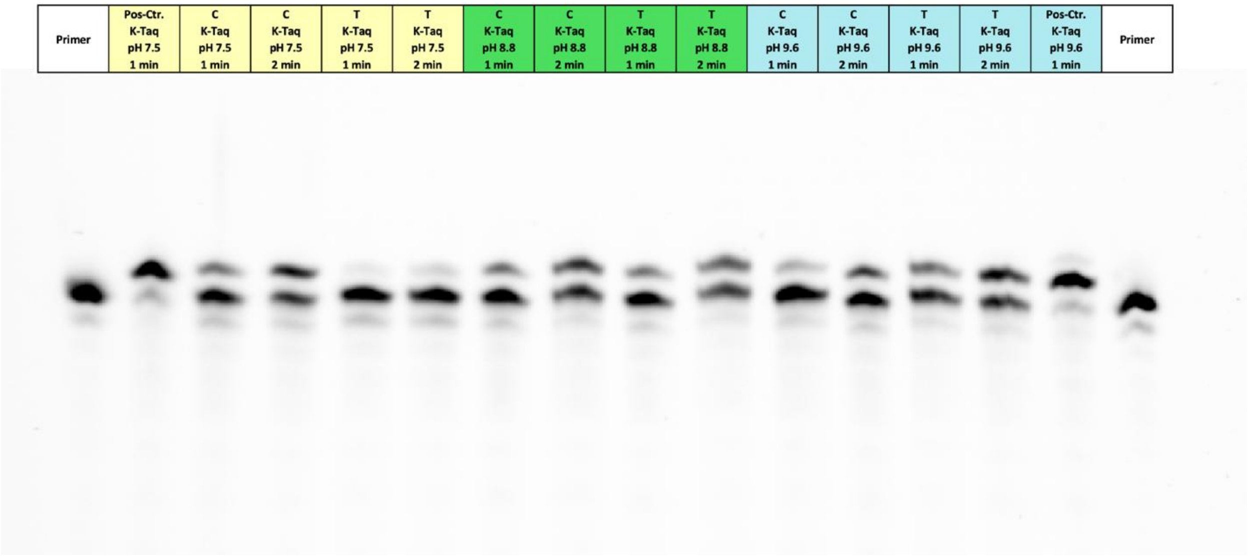

Figure S18: Uncropped image of Figure S4. Urea-PAGE of the incorporation of 2-Amino-DDP triphosphate with KlenTaq. Conditions: 0.5 µM template, 0.25 µM primer, 25 µM dNTP, 0.05 units/µL polymerase, 25 mM Tris-HCl, 40 mM KCl, 5 mM $MgSO_4$, 60 °C.

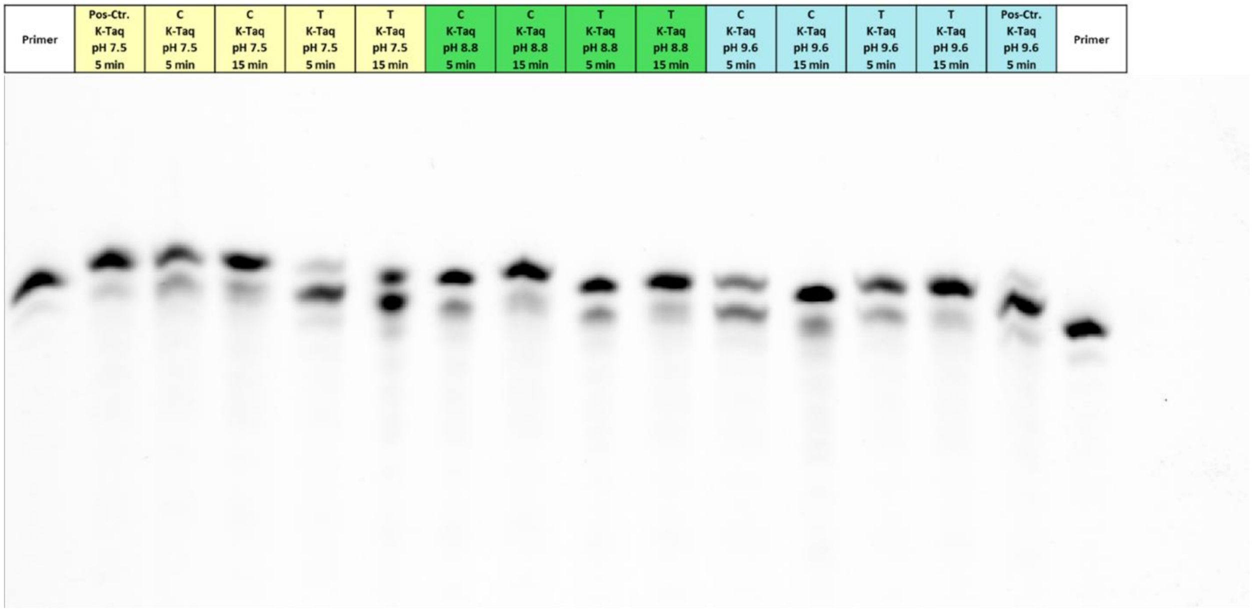

Figure S19: Unedited image of Figure S5. Urea-PAGE of the incorporation of 2-Amino-DDP triphosphate with KlenTaq. Conditions: 0.5 µM template, 0.25 µM primer, 25 µM dNTP, 0.05 units/µL polymerase, 25 mM Tris-HCl, 40 mM KCl, 5 mM $MgSO_4$, 60 °C.

Abbreviations

2-AP	2-aminopurine
A	adenine
Ac	acetyl
ACN	acetonitrile
C	cytosine
COSY	correlated spectroscopy
DCM	dichloromethane
DDP	3,7-dideaza purine
ddNTPs	dideoxyribonucleoside triphosphates
DNA	deoxyribonucleic acid
dNTPs	deoxyribonucleoside triphosphates
DTT	dithiothreitol
EDTA	ethylenediaminetetraacetic acid
eq.	equivalents
EtOH	ethanol
FAM	6-carboxyfluorescein
G	guanine
HMBC	Heteronuclear multiple bond correlation

HPLC	high performance liquid chromatography
HRMS	high resolution mass spectrometry
HSQC	heteronuclear single quantum coherence
LC/MS	liquid chromatography/mass spectrometry
MeOH	methanol
NMR	nuclear magnetic resonance
PCR	polymerase chain reaction
ppm	parts per million
RNA	ribonucleic acid
rt	room temperature (typically 20-25 °C)
T	thymine
TEA	triethylamine
TFA	trifluoroacetic acid
TLC	thin layer chromatography
TRIS	tris(hydroxymethyl)aminomethane
